# Potential effects of the discharge of wastewater treatment plant (WWTP) effluents in benthic communities: evidence from three distinct WWTP systems

**DOI:** 10.1007/s11356-024-33462-z

**Published:** 2024-05-06

**Authors:** Carlos Silva, Joana Isabel Santos, Tânia Vidal, Susana Silva, Salomé Fernandes Pinheiro Almeida, Fernando José Mendes Gonçalves, Nelson Abrantes, Joana Luísa Pereira

**Affiliations:** 1 CESAM – Centre for Environmental and Marine Studies, University of Aveiro, Campus de Santiago, 3810-193 Aveiro, Portugal; 2 Department of Biology, University of Aveiro, Campus de Santiago, 3810-193 Aveiro, Portugal; 3 GeoBioTec - Geobiociências, Geotecnologias E Geo-Engenharias, University of Aveiro, Aveiro, Portugal

**Keywords:** Wastewater contamination, Ecological status, Phytobenthos, Benthic macroinvertebrates, Sediment contamination

## Abstract

Wastewater treatment plant (WWTP) effluents can be sources of environmental contamination. In this study, we aimed to understand whether effluents of three different WWTPs may have ecological effects in riverine recipient ecosystems. To achieve this, we assessed benthic phytobenthos and macroinvertebrate communities at three different locations relative to the effluent discharge: immediately upstream, immediately downstream and 500-m downstream the effluent discharge. Two approaches were employed: the ecological status classification as defined in the Water Framework Directive (WFD) based on biological indicators; constrained multivariate analysis to disentangle the environmental drivers (physicochemical variables and contaminants, namely metals, polycyclic aromatic hydrocarbons, pharmaceuticals, and personal care products) of ecological changes across the study sites. The results showed inconsistencies between the WFD approach and the multivariate approach, as well as between the responses of macroinvertebrates and diatoms. The WWTP effluents impacted benthic communities in a single case: macroinvertebrates were negatively affected by one of the WWTP effluents, likely by the transported pharmaceuticals (other stressors are essentially homogeneous among sites). Given the findings and the scarcity of consistent evidence on ecological impacts that WWTP effluents may have in recipient ecosystems, further research is needed towards more sustainable regulation and linked environmental protection measures.

**Supplementary Information:**

The online version contains supplementary material available at 10.1007/s11356-024-33462-z.

## Introduction

Despite rivers and streams being amongst the most endangered ecosystems worldwide (Gozlan et al. 2019; Sumudumali and Jayawardana 2021), they constitute significant hotspots of biodiversity (Román-Palacios et al. 2022) and are a substantial source of accessible freshwater reserves worldwide (Izmailova and Rumyantsev 2016). The disruption of riverine communities can directly translate into socioeconomic impacts driven by negative changes in water services (e.g., purification, storage, provisioning). Adding to the problem is the known difficulty to regulate and restore impacted ecosystems, leading to long-lasting impacts (Pinheiro et al. 2020). In Europe, the management of aquatic resources and their protection/restoration is mostly addressed through Directive 2000/60/EC, the Water Framework Directive (WFD). The WFD changed the focus of water management by adopting an eco-centric perspective (European Commission 2000), towards the achievement of good ecological status in all EU waterbodies (Bunzel et al. 2013). For ecological status (high, good, moderate, poor, and bad) classification purposes, the WFD assessment scheme relies on hydromorphological, chemical, and biological criteria, the overall classification of the water body corresponding to the lowest classification among these criteria (Santos et al. 2021). Biological criteria are based on the use of bioindicator communities that capture long-term effects of stressors thus overcoming for example the limitation of several physical and chemical methods in reflecting instantaneous conditions (Resende et al. 2010).

The use of bioindicator communities is based on the different tolerance that each taxon has to environmental conditions (Blanco and Bécares 2010; Sumudumali and Jayawardana 2021). Benthic macroinvertebrates are amongst the earlier and more commonly used bioindicators of water quality in rivers. Among the favorable characteristics for their use are their ubiquity, diversity, importance in ecosystem functioning, and differential sensitivity to environmental impact across taxa (Manzoor et al. 2021; Santos et al. 2021). Benthic diatoms, used as proxy for phytobenthos, are also a recommended group for the assessment of ecological status of riverine ecosystems under the WFD (Almeida et al. 2014). They are the second most used taxonomic group (after macroinvertebrates) in the evaluation of ecological quality of rivers in Europe (Masouras et al. 2021). Diatoms are fast responding organisms to a variety of stressors, which renders them very sensitive to environmental changes and holders of a pivotal diagnostic potential (Masouras et al. 2021).

Effluents discharged by wastewater treatment plants (WWTPs) can be a source of contaminants potentially affecting biotic communities in recipient ecosystems. Indeed, WWTP effluents have been considered major sources of several contaminant classes of emerging concern (e.g., pharmaceuticals; personal care products) (Fairbairn et al. 2016; Sörengård et al. 2019; Bashir et al. 2020). This occurs because traditional WWTPs have been designed to remove organic matter and disinfect the influent, thus they are not specifically optimized to remove these different contaminant classes that WWTPs now increasingly receive (Stalter et al. 2011; Burdon et al. 2020). The presence of these compounds in treated effluents is hence common (Bai et al. 2018). Effluent-sourced contaminants may negatively impact biological communities (Masseret et al. 1998; Spänhoff et al. 2007; Tornés et al. 2018) and ecosystem functions (Pascoal et al. 2003; Bundschuh et al. 2011; Burdon et al. 2020); the likelihood and severity of these effects increasing as the dilution capacity of the waterway decrease (Tornés et al. 2018). Adding to the problem is the fact that effluents are complex mixtures of contaminants that may interact, potentially comprising synergic increase of their toxic effect beyond toxicity of individual components (Kienle et al. 2019). Besides contamination with xenobiotics and although the main objective of WWTPs is reducing organic pollution, most effluents still bear high organic content (Tornés et al. 2018; van Gijn et al. 2021), which can be an important driver of change in riverine communities, with multiple studies showing that nutrient increase can impact macroinvertebrate (Ortiz et al. 2005; Friberg et al. 2009; Poulton et al. 2015) and diatom communities (Berglund et al. 2015; Tornés et al. 2018).

Under this context, the present study intended to assess whether WWTP effluents can structurally affect macroinvertebrate and diatom communities of recipient riverine ecosystems of three WWTP effluents. We hypothesize that (i) the contaminants discharged through the effluent promote negative structural changes in macroinvertebrate and benthic diatom communities, as well as the decrease of the corresponding ecological status sensu the WFD; (ii) the distance to the effluent entry point dilutes negative effects in communities’ structure and in ecological quality status; and that (iii) the two biological communities may exhibit different responses to a stressor as complex as WWTP effluents; however, the ecological status (as an integrated “endpoint”) indicated by both should converge. The two groups of organisms are different in their ecological context and requirements, which could suggest that they may respond structurally to environmental shifts in different ways. However, the multimetric indices used to determine ecological quality ratios following the WFD bioassessment are calibrated considering the abiotic context (see Santos et al. 2021 for details) and the entailed biotic indices rely on the scoring of relative sensitivity to diffuse organic pollution (assumed generally in the regulatory field as a proxy to sensitivity to pollution in general) by each accounted taxon. In order to appropriately tackle these hypotheses, we monitored the benthic macroinvertebrate and diatom communities within riverine ecosystems receiving the effluents of three WWTP of different dimensions and holding different treatment processes. The sampling design included samples collected upstream and downstream of the effluent discharge (immediately after the effluent outfall and 500 m downstream the outfall), allowing insights on both the contribution of the effluent to community changes and the potential dilution effects imposed by the flow of the recipient waterways. It is noteworthy that the effluents are not the single stressor pressuring the studied recipient ecosystem, thus the upstream sites are also contaminated (see Silva et al. 2022). However, our study was developed tackling concrete hypotheses in real scenarios, thus applying existent knowledge and evidence (often deriving from sampling in selected sites where contamination gradients could be controlled) to actually existent case studies to understand the additional impact that effluent discharge may have in already degraded ecosystems.

## Material and methods

### Sampling sites and sampling design

Three Portuguese WWTPs were targeted in the present study (WWTPa, WWTPb and WWTPc).

WWTPa serves 160,000 population equivalents from rural and urban settlements, the effluent is discharged after primary treatment (settling and equalization) into a natural sandy creek bearing a low to moderate water flow, and according to the WFD classified as littoral typology (central coastal rivers (APA 2021)); WWTPb serves 50,000 population equivalents in a mostly urban area, treatment includes primary treatment (settling and equalization) and secondary treatment (biological reactor), and the effluent is discharged into a natural creek bearing a moderate to high flow, also classified as a central coastal river (APA 2021); WWTPc serves 700,000 population equivalents in a dense urban area, the influent undergoing primary treatment (settling and equalization) and secondary treatment (biological reactor, settling, and biofiltration); the effluent is discharged into a semi-modified creek (partial embankment and water-control ditches) bearing a low to moderate water flow and with heterogeneous substrate ranging from muddy to structured gravel-cobble areas, which belongs to the sedimentary deposits of the Tagus and Sado rivers regarding river typology (APA 2021). Three sampling points were selected for each waterway receiving the effluent from each WWTP: immediately upstream the effluent discharge (UPa, UPb, and UPc), to establish the background for comparisons allowing the identification of effluent effects in communities (the effluents are not the single pressure over the studied systems); immediately downstream the effluent discharge (D1a, D1b, and D1c), expecting to capture the strongest effects of effluent contaminants in the benthic communities; 500 m downstream the effluent discharge (D2a, D2b, and D2c), allowing an insight on the spatial dilution of putative effects in communities.

### Environmental characterization

The three effluents, as well as the water and sediments of the recipient waterways, were characterized in a previous study, including contaminants of emerging concern (Silva et al. 2022); a summary of this effort is provided in Table S1. The data characterizing water and sediments at each sampling site were assembled in four matrices: (i) the physicochemical (PC) matrix, collecting several water quality parameters, flow speed, depth, and sediment quality parameters; (ii) the metals (M) matrix, collecting the concentrations of 44 metals and metalloids quantified in sediments; (iii) the pharmaceuticals and personal care products (PPCP) matrix, collecting the concentrations of 12 compounds of this class as found in sediments; (iv) the polycyclic aromatic hydrocarbons (PAH) matrix, which includes the concentrations of 16 most concerning compounds as found in sediments (see Table S1). PPCPs were the contaminants with a clearer association with effluents discharge, with 35, 45, and 42 being quantified in the effluent from WWTPa, WWTPb, and WWTPc, respectively; no PPCPs were quantified in WWTPa, 1 was quantified in WWTPb, and 12 were quantified in WWTPc.

### Collection and processing of biological samples

Sampling of the communities was carried out according to the national guidelines complying with the WFD bioassessment scheme for phytobenthos (INAG 2008a) and benthic macroinvertebrates (INAG 2008b). Phytobenthos was collected at each sampling site by scrubbing and washing five pebble-to-cobble (5–15 cm) sized stones from a 50-m stretch to ensure coverage of the different microhabitats and shading conditions. Samples were preserved with a Lugol’s iodine solution, transported in an ice cooler box to the laboratory, where they were stored in a refrigerator protected from light until further processing. Afterwards, samples were cleaned using nitric acid and potassium dichromate to remove organic matter, followed by repeated washing with distilled water. A drop of clean sample was allowed to dry at room temperature on a cover slip which was mounted on a glass slide with Naphrax® to obtain permanent slides. Macroinvertebrate communities were sampled at each site through kick-sampling five small transects, using a standard hand-net (500-μm pore size; square frame 0.30 × 0.30 m), ensuring a proportional representation of the site’s microhabitats. Collected samples were placed in air-tight plastic containers and preserved with 80–90% ethanol for transportation to the laboratory. Therein, samples were washed through a 500-μm sieve, and the organisms retained in the sieve were sorted out. These were then stored in 70% ethanol until identification.

### Identification of organisms and ecological quality metrics

Taxonomic identification of diatoms (to the species level) was carried out using the slides prepared beforehand, under a light microscope (Leica model DM6 B) with differential interference contrast (DIC) imaging. At least 400 valves were counted in each slide and identified using international floras (Krammer and Lange-Bertalot 1986, 1988, 1991a, 1991b; Cantonati et al. 2017). All preserved benthic macroinvertebrates were counted and identified to the family level (except for Oligochaeta, Ostracoda, Hydracarina) using general and taxon-specific identification keys (Macan 1959; Elliott et al. 1977; Pattée and Gourbault 1981; Richoux 1982; Tachet et al. 2000; Sundermann et al. 2007; Serra et al. 2009). Richness, diversity and equitability (S, H’, and J’) were calculated for each sample regarding either diatoms or macroinvertebrates.

For the determination of the ecological status by diatom communities in compliance with the WFD, the biotic index IPS (Indice de Polluosensibilité Spécifique; CEMAGREF 1982) was calculated using the OMNIDIA software (version 5.3—Lecointe et al. 1993) (Eq. 1). Finally, the ecological quality ratio (EQR) was calculated taking into account the reference benchmarks for the river typology of each sampled site (see the “Sampling sites and sampling design” section).

For macroinvertebrates, three standard biotic indices were calculated in compliance with the WFD: (1) the average score per taxon, IASPT, which is derived from IBMWP (Alba-Tercedor 1988) (2) *log* (sel. ETD + 1) or *log* (sel. EPTCD + 1) depending on the river typology, which is the logarithm of the sum of the abundance of selected sensitive taxa based in their autoecology (Heptageniidae, Ephemeridade, Brachycentridae, Goeridae, Odontoceridae, Limnephilidae, Polycentropodidae, Athericidae, Dixidae, Dolichopodidae, Empididae, Stratiomyidae); (3) number of EPT taxa (taxa belonging to orders Ephemeroptera, Plecoptera, and Trichoptera). Although offering information per se, these biotic indices were essentially determined for normalization (to corresponding reference values; APA 2021) and further integration to calculate the multi-metric index IPtI_s_ (Eq. 2).

In order to determine the EQR (that are then convertible in ecological quality statuses) of each sampled site based on its macroinvertebrate community, the values obtained for IPtI_S_ were normalized by dividing by the corresponding type-specific reference value (APA 2021)

1 $$IPS=\frac{\sum_{j=1}^{n}{a}_{j }{s}_{j {v}_{j}}}{\sum_{j=1}^{n}{a}_{j }{v}_{j}}$$

where ${a}_{j}$ is the relative abundance of species $j$; ${s}_{j}$ is the sensitivity of species $j$; ${v}_{j}$ is the indicator score of species $j$

2 $$IPt{I}_{S}=\left(0.4*S\right)+\left(0.2*EPT\right)+\left(0.2*IASPT-2\right)+(0.2*Log\left(Sel.EPTCD+1\right))$$

### Multivariate data analysis regarding community structure assessment

As a pretreatment applied to each group of environmental variables characterizing the sites in terms of chemical contamination (three matrices: M, PPCP, PAH; see the “Environmental characterization” section), a Pearson correlation matrix was built, allowing the identification of highly correlated variables (Pearson’s *r* ≥ 0.8; Table S2). This approach served two main purposes: (i) prevent recognizable redundancy in constrained multivariate analysis a priori; (ii) decrease the number of environmental variables in the analysis to ranges fulfilling pre-defined requirements, thus ensuring that the number of variables was smaller than the number of species included in the community datasets. Variables found highly correlated were used to generate representative omnibus variables integrating the abiotic matrix (PAHs, PPCPs, and metals within the PAH, PPCPs, and M matrices, respectively) by averaging among corresponding values. All variables of the environmental matrix (including PC) were then standardized prior to any further analysis to prevent scale-related effects in subsequent analyses.

The multivariate analysis using the community (species abundance matrices available in Table S3) and environmental matrices was carried out using the CANOCO software (Ter Braak and Šmilauer 2002). While in Silva et al. (2022) environmental gradients were confirmed (despite the low number of sites assessed), herein constrained ordination methods were used to explore if environmental gradients could explain biological gradients, then interpreting on whether the environmental context imposed by the effluent discharge induces changes in the structure of benthic communities within each WWTP, and on whether there is a differential response of communities among the three case-study WWTPs. Although species abundance generally tends to follow a unimodal response to environmental gradients (Jongman et al. 1995), diagnosis detrended correspondence analysis (DCA) revealed a short gradient in the distribution of the macroinvertebrate communities (length of gradient = 1.83 s.d.). As such and according to ter Braak and Prentice (1988), redundancy analysis (RDA) was selected to address the putative constraints imposed by the environmental context to these communities. The same rationale applied for the selection of canonical correspondence analysis (CCA) to address the environmental constraints of diatom distribution, provided that a larger length of gradient of 5.71 s.d. was found in the corresponding diagnosis DCA (Ter Braak and Prentice 1988; Ter Braak and Šmilauer 2002). Constrained multivariate analysis (RDA for macroinvertebrates and CCA for diatoms) was run considering inter-species distances separately for macroinvertebrates and diatoms and using all variables available from all environmental matrices after pre-treatment (see above) jointly as the constraining dataset. A manual forward selection procedure was applied to the environmental variables, to reduce the model to non-redundant variables, significantly explaining the ecological gradients (Monte Carlo permutation tests; *p* ≤ 0.05).

## Results and discussion

The saprobic index (Kolkwitz and Marsson 1902), one of the earliest biotic indexes for water quality assessment, was developed to characterize the ecological effects of organic pollution. However, nowadays domestic originated sewage contains a plethora of new contaminants, including those commonly known as contaminants of emerging concern (CEC), and some tend to bypass WWTP treatment, being released in the environment (Johnson 2019), making the assessment of effluent-derived ecological effects more challenging but likely more needed than ever, especially considering that potential effects are not fully understand (Weitere et al. 2021). Herein, potential effects of effluent-associated contamination of riverine ecosystems were assessed in the field and focusing on benthic communities. The environmental gradients involved in the three case studies have been explored in detail by Silva et al. (2022) based on principal component analyses, thus only a brief summary is provided herein with the support of Table S1 for a clearer interpretation of the subsequent sections. Note in addition that the effluent load in contaminants is also characterized in Silva et al. (2022), yet sediments are the main focus herein.

Concerning general physicochemical characterization, the sites were similar in general (Table S1). Flow speed was lower by one order of magnitude or more in samples D2a and D2b compared to the other records within the corresponding WWTPs. There were distinguished increases in conductivity in sites D2b and D2c, and reduction in dissolved oxygen in sites D1c and D2c, compared to the rest of the sites. Nutrient (N and P) levels, which constrain periphyton communities, are generally low and their variation within WWTP is low as well. Sediment characterization (organic matter and granulometry) showed low variability among sites within WWTP.

Sediments were focused to characterize contamination in the sites rather than the water. This was an assumed option considering that the targeted recipient ecosystems are lotic, thus any picture retrieved from water in terms of contaminants profile would be ephemeral and dependent on the discharge schedules. Concerning the quantified chemical contaminants and regardless of their class, the sediments in WWTPc were the most heavily contaminated compared to the other two WWTPs (Silva et al. 2022; Table S1). Metal concentrations tended to be similar among samples within WWTP, except for D1b, where Ba, Pb, and Eu were found at notably higher concentrations than in UPb and D2b, the gradients showing that the effluents have mild to no role as a relevant source of metals in the studied systems (Table S1). For PAHs contamination, most samples have similar concentrations within the same WWTP, except for sample UPc that bears approximately doubled PAH concentrations compared to the other WWTPc; this exempts the effluents from a major role as PAH sources, although it has been confirmed that WWTP effluents can be a source of PAHs (Mojiri et al. 2019). Urban water bodies, which are the case of the stream receiving the WWTPc effluent, are more susceptible to diffuse contamination from traffic compared to those standing in rural areas, promoting PAH input in waterbodies through runoff (Ellis and Mitchell 2006). Indeed, in the vicinity of UPc, notably higher traffic levels in a major road are observed compared to the WWTPc sites downstream the effluent discharge (500 m and 1 km apart UPc and clearly more distant to major roads than UPc), and also compared to other studied WWTPs. PPCPs were only detected in samples from WWTPb (caffeine only in D2b; trimethoprim in UPb and D1b) and WWTPc, and were the single class where differences among sites within WWTP could be noticed, which renders this class an increased relevance in the present study. Twelve PPCPs were detected and quantified in samples downstream the WWTPc effluent discharge, suggesting that the effluent contributed to the sediment load regarding these contaminants, and caffeine was quantified in all WWTPc samples (Table S1). Caffeine can be commonly used as a tracer of sewage presence, and its presence in sample UPc could signal illegal sewage discharge as hypothesized by other authors, e.g., Cantwell et al. (2016) and Paíga et al. (2019).

There are studies in which WWTP effluents were shown to impact biological communities, including macroinvertebrates (Aristone et al. 2022; Enns et al. 2023; Peschke et al. 2019) and diatoms (Tornés et al. 2018; Chonova et al. 2019), although the extent of the impact is dependent on many factors—e.g., Dyer and Wang (2002) reported differences when comparing impacts caused by urban and rural originated effluents. These suspected impacts were the basis of our first study hypothesis, which was addressed using the WFD bioassessment scheme towards ecological status classification and the structure of corresponding macroinvertebrate and diatom communities; and to our related second study hypothesis, concerning dilution effects as distance to the effluent outfall increases, both explored in the “Response of macroinvertebrate communities to the effluents” and “Response of phytobenthos to the effluents” sections.

### Response of macroinvertebrate communities to the effluents

Ecological metrics and indices specifically targeting the macroinvertebrates community (Table 1) suggest that the ecosystems receiving the effluents from WWTPa and WWTPc are under stress, while the picture is not negative in WWTPb, particularly downstream the effluent discharge. In WWTPa and WWTPc, the ecological status was worse (poor or bad) after the effluent discharge point when compared to the corresponding upstream sites, which were classified as moderate and poor, respectively. The corresponding ecological metrics are generally consistent with this pattern provided their integration in the multi-metric indices used to determine ecological quality ratios that then define ecological status classification. These trends support our main hypothesis but did not support the second hypothesis as those impacts were not diluted with increasing distance to the effluent entry point—worse ecological status was found in D2a (bad) than D1a (poor), and the same ecological status (Bad) was found for D1c and D2c. Hydrodynamic factors are very important for sedimentation of sewage-sourced suspended particulate matter (Cabral and Martins 2018), and in D2a, flow speed decreases noticeably (Table S1). Flow reduction may increase the sedimentation of particulate matter with adsorbed contaminants (Khan et al. 2016), driving changes in macroinvertebrate communities (Juvigny-Khenafou et al. 2021), and eventually contributing to the decrease in ecological status; it can also be per se a natural driver of changes in macroinvertebrate communities (Juvigny-Khenafou et al. 2021). In WWTPb, the effluent does not seem to negatively affect benthic macroinvertebrate metrics. Richness increased from UPb to D2b; nevertheless, diversity was similar in both samples and slightly higher in sample D1b. In fact, for this case, the worst classification in terms of ecological quality based on the macroinvertebrate communities was found for the sample upstream the effluent discharge (moderate), which is contrary to the trend found for the other two WWTPs and opposed to our hypotheses that effluents have a negative effect in the biota of recipient riverine ecosystems diluting with increased distance to the discharge. It should be noted that nutrient enrichment tends to increase autotrophic biomass (Dodds 2006) that can cascade in organic matter flow shifts with implications on macroinvertebrate community composition (Cross et al. 2007), and that macroinvertebrates often attain higher densities in mildly eutrophicated streams (Friberg et al. 2011). This macroinvertebrate enrichment phenomena downstream effluent discharge was verified by Dyer and Wang (2002) for WWTPs located in areas with population densities < 500 per square mile, as it is the case of WWTPb. A contribution of the effluent to promote these favorable scenarios downstream the outfall could be hypothesized. However, N levels, P levels, or sediment organic matter were essentially similar among sites within WWTPb (Table S1), which suggests that other environmental factors should have contributed to explain the water quality status variation found therein.

**Table 1:** Community composition metrics, multi-metric indices recommended for the calculation of ecological status, and the ecological status of each sample calculated considering either benthic macroinvertebrates or phytobenthos

	WWTPa			WWTPb			WWTPc		
	Up	D1	D2	Up	D1	D2	Up	D1	D2
***Macroinvertebrates***									
Richness (S)	8	9	3	11	15	18	13	4	4
Diversity (H’)	0.77	1.48	0.32	1.14	1.54	1.03	0.99	0.58	0.31
Equitability (J’)	0.37	0.67	0.29	0.47	0.57	0.36	0.39	0.42	0.22
EPT	0.25	0.13	0	0.25	0.38	0.63	0.20	0	0
IPtI_S_	0.38	0.32	0.03	0.52	0.56	0.73	0.42	0.09	0.10
EQR	0.39	0.33	0.03	0.54	0.57	0.74	0.40	0.09	0.10
Ecological status	Moderate	Poor	Bad	Moderate	Good	High	Poor	Bad	Bad
***Diatoms***									
Richness (S)	25	24	14	22	22	26	13	9	14
Diversity (H’)	1.9	2.18	2.19	2.93	2.85	3.15	2.06	1.52	2.97
Evenness (J’)	0.41	0.48	0.57	0.66	0.64	0.67	0.56	0.48	0.78
IPS	17.30	17.7	16.10	14.90	13.60	14.90	2.50	2.00	5.80
EQR	0.99	1.02	0.93	0.86	0.78	0.86	0.15	0.12	0.35
Ecological status	High	High	Good	Good	Good	Good	Bad	Bad	Poor

**Fig. 1:**
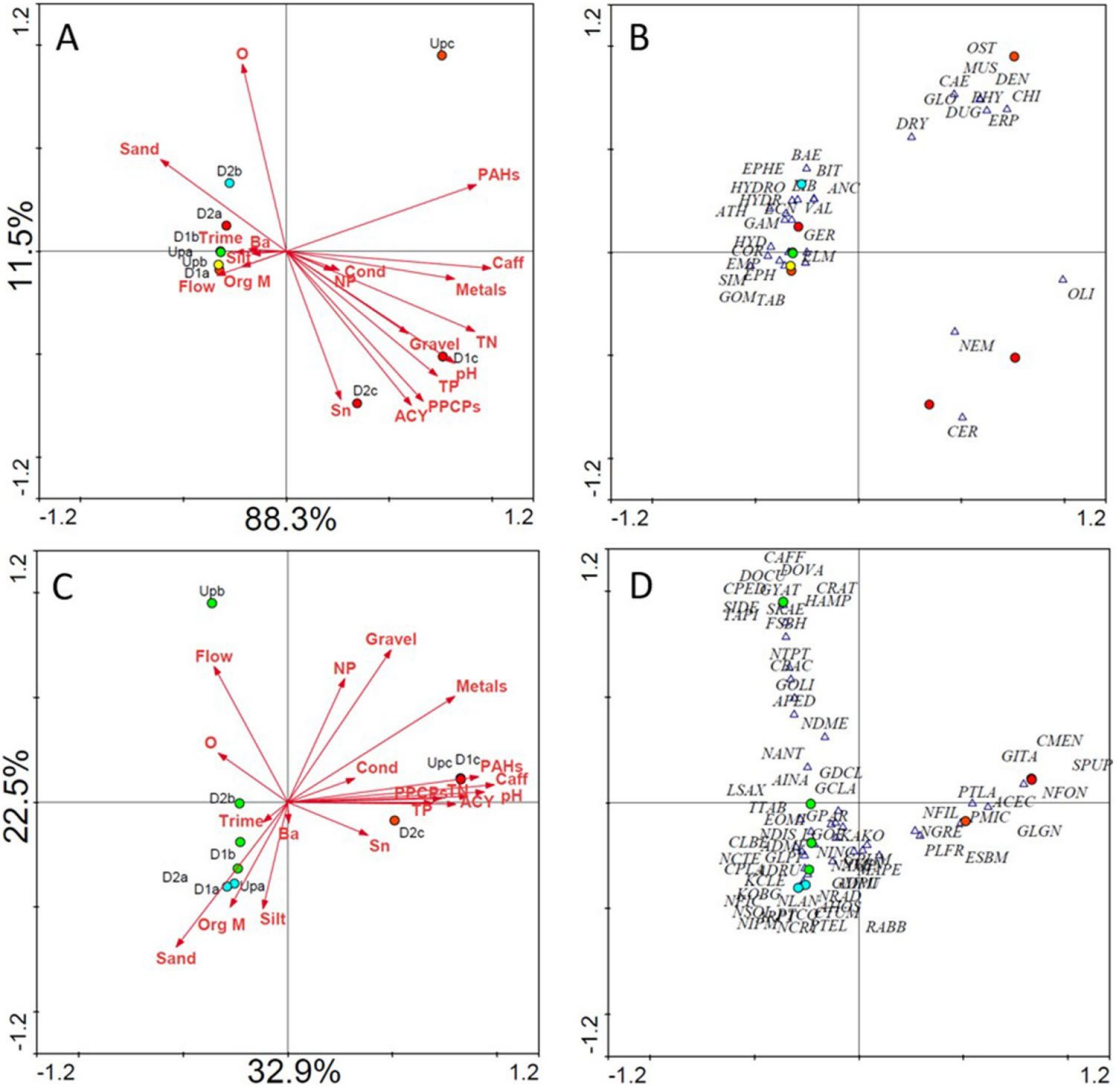
Ordination biplots, RDA for macroinvertebrate communities (**A**, **B**) and CCA for phytobenthos (**C**, **D**) representing the constraints imposed by all non-colinear environmental variables (see the “Multivariate data analysis regarding community structure assessment” section; table S2). Site scores (circles) and environmental gradients (arrows) are represented in the left-hand plots, while species (triangles) are represented with sites in the right-hand plots for improved readability. Each site was colored according to its ecological status using the color code recommended by the WFD directive, blue-high; green-good; yellow-moderate; orange-poor; red-bad

The outcome of multivariate community structure analysis was not consistent with the outcome of the WFD approach described above. This is an immediate conclusion achieved upon the evidence of poor, non-significant correlation between EQR values for each site and their RDA scores, regardless of the axis (axis 1 or 2) or model (full or reduced) focused (Table S5). Physicochemical variables, such as nutrients, pH or coarser sediment, metals (as an omnibus variable), ACY and PAHs (as an omnibus variable), caffeine and PPCP (as an omnibus variable) seem to drive a clear separation of WWTPc with distinguished community structure compared to the other two WWTPs, while explaining 99.8% of the bidimensional distribution of species (Fig. 1A). All samples from WWTPa and WWTPb were clustered together with low levels of the referred environmental variables (Fig. 1A). Sensitive taxa such as Athericidae (ATH), Libellulidae (LIB), and Ecnomidae (ECN) are associated with samples from WWTPb, while both samples from WWTPa and WWTPb have relatively high abundances of moderately tolerant species like Simuliidae (SIM), Baetidae (BAE), and Hydrobiidae (HYD). Sample UPb contained individuals from families that are not present in any other sample, namely Ostracoda (OST), Muscidae (MUS), Dendrocoelidae (DEN), Dugesiidae (DUG), Erpobdellidae (ERP), and Glossiphoniidae (GLO), while the other samples from WWTPc are clearly dominated by Chironomidae (CHI) and Oligochaeta (OLI) (Fig. 1B). WWTPc sites are associated to higher values of nearly all environmental variables, clearly denoting higher contamination by nutrients and xenobiotics; dissolved oxygen levels were also lower in WWTPc samples downstream the effluent discharge compared to all other samples (Fig. 1A). This overall scenario is consistent with the association of tolerant taxa to WWTPc sites in general (e.g., Physidae (PHY); CHI), then the association of OLI, Nemathelmintha (NEM) and Ceratopogonidae (CER) specifically to sites D1c and D2c, which are taxa typically tolerant to low oxygenation (Pardo and García, 2016; Etemi et al. 2020). Indeed, oxygen levels seem to be an important factor constraining the separation of UPc from D1c and D2c along the second axis (explaining 11.5% of the taxa distribution), while contamination by nutrients and xenobiotics, read mostly along the first axis, did not distinguish these three sites of WWTPc.

**Fig. 2:**
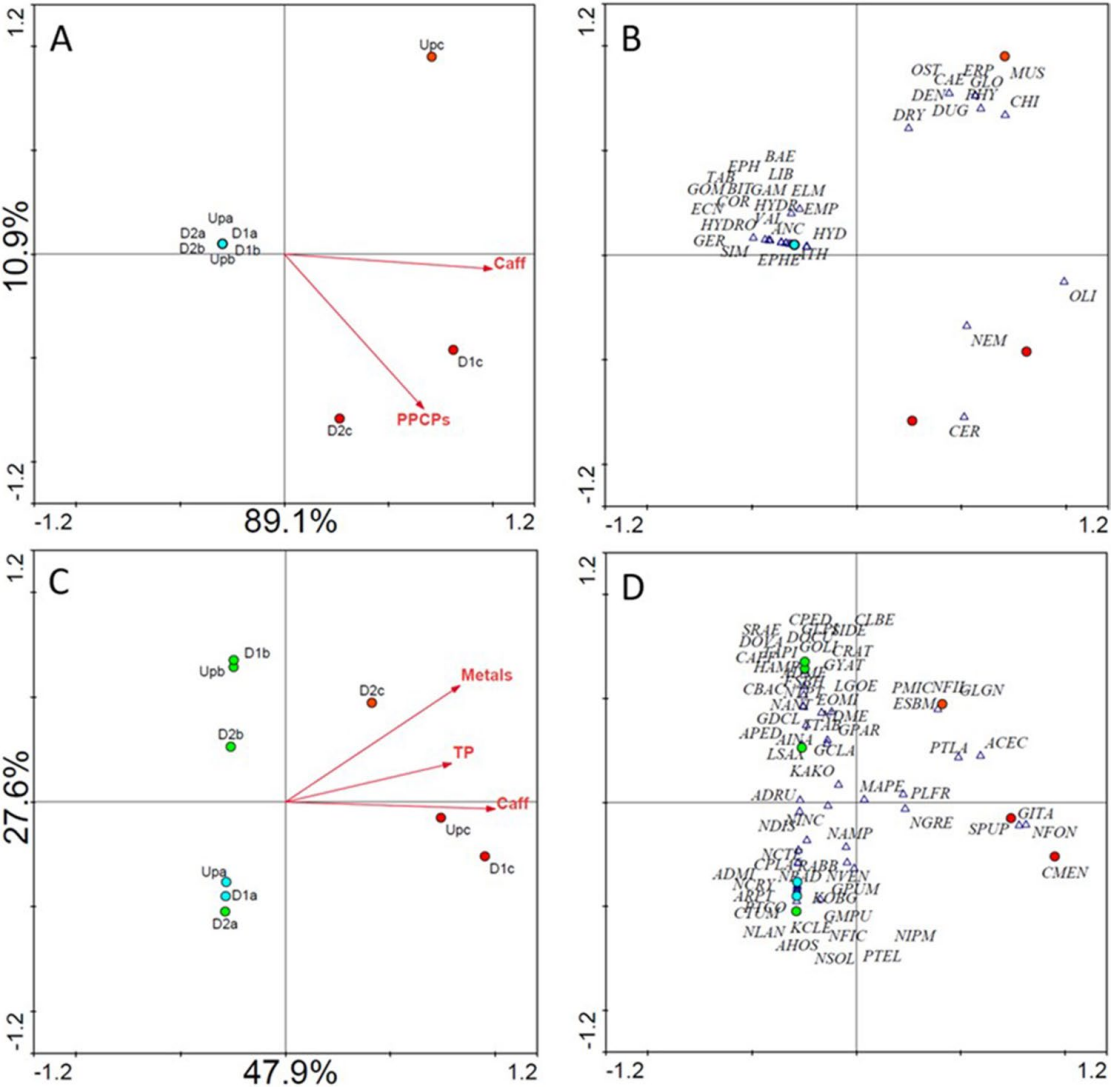
Ordination biplots, RDA for macroinvertebrate communities (**A**, **B**) and CCA for phytobenthos (**C**, **D**) representing the reduced model addressing constraints imposed by environmental variables significantly explaining taxa distribution after a forward selection procedure (unrestricted Monte-Carlo permutations) apart from those a priori excluded as per redundancy (see “Multivariate data analysis regarding community structure assessment” section). Site scores (circles) and environmental gradients (arrows) are represented in the left-hand plots, while species (triangles) are represented with sites in the right-hand plots for improved readability. Each site was colored according to its ecological status using the color code recommended by the WFD directive, blue-high; green-good; yellow-moderate; orange-poor; red-bad

On the other hand, as the RDA model was reduced to the variables significantly explaining taxa distribution following a forward selection procedure (Monte Carlo permutation tests, *p* < 0.05), the relevance of caffeine (*F* = 47.876, *p* = 0.0020) and the omnibus PPCPs variable (*F* = 5.735, *p* = 0.0560) became apparent (Fig. 2A) without changing the explanatory power of the model. Dissolved oxygen, which was one of the variables that could apparently explain distribution across the second axis by interpreting Fig. 1A, was found to not significantly explain the taxa distribution (Table S4); thus, it was not statistically selected to the reduced model. PPCPs as an omnibus variable was thus clearly evidenced as the variable explaining the separation between UPc and the two downstream sites in WWTPc, while the global trends in the samples distribution remains the same in the reduced model (Fig. 2A, B) when compared to the full model (Fig. 1B): samples from WWTPa and WWTPb (now indistinguishable) separated from the WWTPc samples influenced by higher concentrations of caffeine, already shown to negatively affect macroinvertebrates (Mustard 2014) and omnibus PPCPs (several known to induce negative effects in different macroinvertebrate species in, e.g., Santos et al. 2010; Fong and Hoy 2012; Minguez et al. 2014; Bose et al. 2022). Furthermore, no differences in the macroinvertebrates community composition are noticeable from Figs. 1B to 2B: the most sensitive taxa as mentioned above remain mainly associated with WWTPb (ATH, LIB, ECN), and this is also valid for most taxa in the left side of Fig. 2B. Similarly, the taxa that seem most influential in separating samples D1c and D2c from UPc remain CER, NEM, and OLI, with PHY only detected in sample UPc and Caenidae (CAE) holding doubled abundance in UPc compared to other samples. In fact, taxa associated with samples D1c and D2c (NEM; OLI; CER) are tolerant to organic pollution and indicators of low ecological quality (Höss et al. 2006; Johnson et al. 2019); some of the taxa associated with UPc are noticeably more sensitive, e.g., DUG or CAE, while others are of median sensitivity (ERP, GLO) (Paisley et al. 2014).

The multivariate approach suggests that changes in the macroinvertebrate communities in WWTPc are effluent-driven, supporting our first hypothesis and enlightening that the contaminants carried by the effluent (Silva et al. 2022) affect these communities; contributing to this role are specifically PPCPs. Metals and PAHs, as well as the other monitored physicochemical variables, seem to have a negligible role, which is consistent with the environmental gradients in this system as found by Silva et al. (2022). The multivariate approach also showed that the hypothesized dilution of effects in communities as distance to the effluent discharge increases was not verified in WWTPc, and that neither the noxious role of the effluent nor the dilution of effects was identified in the other two case studies. This evidence is inconsistent with the outcome provided by the ecological quality classification using macroinvertebrates as bioindicators regarding WWTPa, which suggests that the WFD approach, using multi-metric indices calibrated for organic pollution, i.e., nutrients (Sandin and Hering 2004; Van de Bund 2009) might not be appropriately tuned to current scenarios of aquatic contamination as already claimed by several authors (Brack et al. 2017; Kortenkamp et al. 2019; Büttner et al. 2022; Weisner et al. 2022). It has been stated that impoverished macroinvertebrate communities, such as those expected in urban streams like that sampled in WWTPc (note the lower taxa diversity associated to WWTPc sites in Figs. 1B and 2B), tend to be less responsive to stressors because the community is already constituted by tolerant taxa (Grantham et al. 2012). However, WWTPc provided converse evidence (sensitivity to the effluent discharge), inherently questioning the definition of what should be considered a tolerant taxon and subsequently the reliance in indices that may reflect outdated contamination scenarios to assess ecological quality.

### Response of phytobenthos to the effluents

In WWTPa, the ecological status was found to be high or good taking in consideration the diatom community (Table 1), the decrease from high (UPa and D1a) to good (D2a) being possibly related to the decrease in the relative abundance of *Achnanthidium minutissimum*, which has the highest score for sensitivity in the IPS, while more tolerant species such as *Navicula veneta* and *Navicula lanceolata* were present in D2a but were absent from UPa and D1a; *Amphora pediculus* and *Navicula gregaria* which are moderately tolerant, showed double the abundance in D2a compared to UPa and D1a (see Table S3). Reduced flow in D2a compared to UPa and D1a may concur to explain this decrease in ecological quality signaled by diatoms (Table S1) since lower flow tends to be disadvantageous to diatoms by decreasing the oxygen available, then shaping the assemblages (Wang et al. 2018). Similar ecological metrics were observed among sampling sites within WWTPb, all classified as good regardless of the distance to the effluent discharge. In WWTPc, all sites are clearly under remarkable environmental stress as all were found to have bad or poor ecological status based on phytobenthos, improving from bad upstream (UPc) and immediately after the effluent discharge (D1c) to poor in D2c. This improvement in ecological status does not seem grounded in the measured physicochemical parameters and quantified contaminants, as they are rather similar among those two samples downstream the effluent discharge (Table S1). Based on the ecological status classification, our hypothesis that the effluent could drive changes in diatom communities was refuted for all case studies, and consequently, there was no context to assess on whether effects dilute with distance to effluent discharge (derived second hypothesis).

The multivariate approach agreed in general with the WFD approach as retrieved when correlating EQR values with sample scores in CCA models (as confirmed by the strong, significant correlations between EQR values and axis 1 scores; see Table S5) but was naturally more informative as to the environmental factors constraining the diatom communities in different sampling sites. As the full environmental matrix was tested, the separation of WWTPc sites from the other sites along the first CCA axis is clear in the right side of Fig. 1C, suggesting that communities therein are shaped by higher nutrient levels, higher PAH, metal, and PPCPs burden. This trend was kept when the reduced model was applied to constrain communities distribution (Fig. 2C), suggesting that the most relevant drivers of community structure at WWTPc sites are increased levels of nutrients (TP), metals and caffeine and remarkably achieving a better explanation of the phytobenthos distribution (75.5% vs. 55.4% of the variance explained by the full model). PPCPs and PAHs as omnibus variables were excluded as significant explanatory variables, but their relevance in explaining taxa distribution cannot be definitively ruled out. The omnibus PPCPs variable was found to significantly explain phytobenthos distribution when the variables were tested before model reduction (Table S4), but caffeine explained more of the model variance (87% vs. 60% for PPCPs; Table S4) and after the inclusion of caffeine in the model during the forward selection procedure (see the “Material and methods” section for details), PPCPs no longer significantly contribute to further reducing the model uncertainty. While PPCPs, but not metals, are confirmedly sourced by the effluent in WWTPc (Silva et al. 2022), the hypothesis that the discharge of the effluent negatively affects phytobenthos communities was not strongly supported. In WWTPc, the majority of the diatom species have low ecological indicator values, i.e., low ecological requirements, regardless of the site that they are associated to *Cyclotella meneghiniana* (CMEN), *Sellaphora pupula* (SPUP), *Eolimna subminuscula* (ESBM), *Gomphonema lagenula* (GLGN), and *Nitzschia filiformis* (NFIL). Although the information available is very scarce, some of these species have been shown to be tolerant to pharmaceuticals. Gomaa et al. (2021) found that CMEN and NFIL were tolerant to the high concentrations of both paracetamol and ciprofloxacin, and Andreozzi et al. (2004) reported CMEN tolerance to the antibiotic amoxicillin. The separation of D2c from UPc and D1c that clustered together (Figs. 1C and 2C) is consistent with the improvement noticed in the ecological status noticed from UPc and D1c to D2c (Table 1). Indeed, species associating to D2c, such as as *Planothidium lanceolatum* (PTLA) and *Achnanthidium exiguum* var. *constrictum* (ACEC) are species categorized of median to high sensitivity to diffuse organic pollution, with scores of 4.6 and 4.0 respectively in the IPS scoring system (Lecointe et al. 1993). Still, and apart from TP, which has proven effects in phytobenthos distributions (Pandey et al. 2018; Wang et al. 2019), the stressors used in the IPS scoring system are distinct from those significantly constraining phytobenthos communities in our study, i.e., metals and caffeine (Fig. 2C).

The full CCA model resulted in a separation of UPb from the other two samples (D1b and D2b) across the second axis (Fig. 1C), which could indicate that the effluent discharge has a role in shaping the diatom communities in WWTPb. Higher flow, oxygen concentrations, coarser sediments and higher levels of metals (omnibus variable) are the environmental variables apparently driving this separation (He et al. 2011). Indeed, the distribution of phytobenthos can be influenced by granulometry, the concentrations of major ions (Potapova and Charles 2003), oxygen (Shibabaw et al. 2021) and flow (Sabater and Roca 1990). However, as the model was reduced to include only significant predictors of the phytobenthos distribution in our study, these variables were not included, and the distance between sites within WWTPb became negligible while lower uncertainty was achieved (Fig. 2C). This rather suggests that the effluent has no significant role in shaping the communities therein. Also, the full model (Fig. 1D) associates species of higher sensitivity to UPb, namely *Cocconeis pediculus* (CPED) and *Cymbella affinis* (CAFF); this association becomes less prominent in the reduced model provided the changes in sites clustering (Fig. 2D). Leira and Sabater (2005) found that CPED and CAFF distribution was mainly justified by physio-geographical factors rather than water chemistry, reinforcing that no major impacts of the effluent in the habitat degradation of the recipient ecosystem can be distinguished in what concerns diatom ecological preferences.

### Macroinvertebrates vs. phytobenthos

Phytobenthic diatoms responded to the effluent differently (often signaling less degraded conditions) compared to macroinvertebrates, in many cases translating into different ecological status classifications (Table 1). This is especially evident for WWTPa, where ecological status was found to be high or good taking into consideration the diatom communities, but moderate or below when focusing on macroinvertebrate communities. Still, there was also no full agreement for the samples of WWTPb. Based on diatoms, ecological status was classified as good in all three samples, while the same evaluation using macroinvertebrate data signaled an increase in ecological status downstream the effluent discharge. In WWTPc, all sites were found to have bad or poor ecological status regardless of the indicator community. However, the ecological status decreased from poor in UPc to bad downstream the effluent discharge (D1c and D2c) when macroinvertebrates were used as indicators, while it improved from bad upstream (UPc) and immediately after the effluent discharge (D1c) to poor in D2c when diatoms were used as indicators. This gives a concise and definitive response to our third research hypothesis, as ecological status of the studied sites using either community did not converge and, as shown above, in some cases the ecological status indicated by macroinvertebrates or diatoms differed by a wide margin.

It is worth further noticing that macroinvertebrates and diatoms agreed in the distinction of communities associated with the most heavily contaminated case study, WWTPc, from communities associated with the other WWTPs (see Fig. 2A, B vs. C, D), but this was the single common trend captured by multivariate analysis. For example, while macroinvertebrates distinguished the role of the effluent in impairing communities in WWTPc supporting our first hypothesis, diatoms did not, with the UPc site holding a similar community compared to D1c; while diatom communities were mildly different among sites with in WWTPa and WWTPb, likely responding to some unmonitored habitat gradient or to metals (note that they separate sites across axis 2 that does not reflect TP and PPCPs/caffeine gradients; Fig. 2C), macroinvertebrate communities were basically similar at all sites in these two WWTPs (see Fig. 2A vs. C).

As for possible explanations for the inconsistency between responses of macroinvertebrates and diatoms, the production of extracellular polymeric substances (EPS) by phytobenthos that some authors argue to have a role in the protection of the community against chemical stressors (Shniukova and Zolotareva 2015; Xiao and Zheng 2016; Gonçalves et al. 2018), seems a good candidate. EPS quantity and characteristics can be regulated by several factors such as oxygen and nitrogen availability, the extent of desiccation, availability of nutrients, pH, temperature, and characteristics of the diatoms themselves namely species and/or strain (Vu et al. 2009; Babiak and Krzemińska 2021). This could have an impact on the reliability of the use of diatom indices for ecological status determination on certain cases, such as in ecosystems affected by contaminants of emerging concern. In this line, the inadequacy of the diatom WFD indices for assessment for example in sites heavily contaminated with metals was already argued (Mendes et al. 2014; Valente et al. 2016). Provided the EPS protection, diatoms within phytobenthos communities can likely better thrive under degrading environmental conditions, including anthropogenic contamination, compared to unprotected communities such as macroinvertebrates.

## Conclusions

The present study was focused on the assessment of putative effects of WWTP effluents in benthic communities of recipient ecosystems, following a previous work characterizing the contaminants load of sediments in these same ecosystems. Our study did not fully support the first research hypothesis regarding the negative impact of effluent discharge on benthic community structure. Consistent support was only obtained for one of our case studies, WWTPc, and specifically when assessing macroinvertebrates. Although the WFD and the multivariate approach did not agree for macroinvertebrates (no correlation between EQR and site scores in RDA), both approaches indicate that macroinvertebrate community structure is negatively affected by the effluent in WWTPc. The ecological status based on macroinvertebrates also decreased with the effluent discharge in WWTPa, but the multivariate approach did not confirm changes in community structure. Our second research hypothesis that putative negative effects of the effluent in communities would dilute in the site furthest for the effluent discharge (~ 500 m), was dependent on the verification of the first hypothesis; as such, in the single valid scenario (there were improvements but with no impairment noticed by the effluent before), i.e., in WWTPc and for macroinvertebrates, dilution effects could not be verified. Macroinvertebrates and diatoms responded differently to the environmental context, thus refuting our third hypothesis and preventing in addition a consistent conclusion on the first one for WWTPc. In particular, herein diatoms did not show negative effects of the effluent in communities by the WFD or the multivariate approach. The largely pictured inconsistency in responses assessed using the WFD and the multivariate approach, as well as between different bioindicator communities, contributes to the suggestions that have been made in the literature on the need to revisit regulatory frameworks, ensuring that ecological gradients, which are altered under current scenarios of contamination in freshwater ecosystems, are accurately captured and effectively managed. Overall, this study did not confirm WWTP effluents as a widely recognizable pressure over recipient riverine ecosystems. However, we addressed three case studies only, which constrain generalizations. Thus, more comprehensive assessment is urgently needed to characterize the potential ecological effects of this contaminant source, in addition to the much more common assessment of the related chemical burden. This information could prove crucial to inform regulators and stakeholders on the actual hazard that WWTP effluents may pose to riverine ecosystems, enabling the development and implementation of more effective management strategies.

### Supplementary Information

Below is the link to the electronic supplementary material.

Supplementary file1 (DOCX 130 KB)

## Data Availability

The data underlying this study are available through the linked supplementary materials.
