# Supplementary material for: Potential effects of the discharge of wastewater treatment plant (WWTP) effluents in benthic communities: evidence from three distinct WWTP systems
Source: Environ Sci Pollut Res Int. 2024 May 6;31(23):34492–506. doi: 10.1007/s11356-024-33462-z (PMC11136724; doi:10.1007/s11356-024-33462-z)
Supplement: Supplementary file 1 — Supplementary file1 (DOCX 130 KB) [file 11356_2024_33462_MOESM1_ESM.docx]

**Supplementary material**

**Potential effects of the discharge of wastewater treatment plant (WWTP) effluents in benthic communities: evidence from three distinct WWTP systems*.***

Carlos Silva, Joana I Santos, Tânia Vidal, Susana Silva, Salomé FP Almeida, Fernando JM Gonçalves, Nelson Abrantes, Joana Luísa Pereira

**Table S1.** Summarized characterization of sampling sites (data collected from Silva et al., 2022). General water and sediment quality parameters are provided first, then quantified concentrations of PPCPs, metallic elements, and PAHs in sediments are given for compounds found in at least one sample analyzed in the present study.

|  | **WWTPa** | | | **WWTPb** | | | **WWTPc** | | |
| --- | --- | --- | --- | --- | --- | --- | --- | --- | --- |
|  | Upa | D1a | D2a | Upb | D1b | D2b | Upc | D1c | D2c |
| Water |  |  |  |  |  |  |  |  |  |
| Flow speed (m/s) | 11.11 | 12.36 | 0.25 | 25 | 27.22 | 9.72 | 7.69 | 9.64 | 8.99 |
| Temperature (ºC) | 18.3 | 17.7 | 17.2 | 17.3 | 18.2 | 17.8 | 28.9 | 24.1 | 23.6 |
| Conductivity (μS/cm) | 398 | 400 | 360 | 505 | 458 | 1435 | 619 | 799 | 1025 |
| Dissolved oxygen (mg/L) | 8.81 | 8.94 | 7.85 | 9.88 | 9.46 | 9.72 | 12.26 | 5.30 | 5.42 |
| Oxygen saturation (%) | 93.9 | 93.7 | 81.1 | 102 | 100.3 | 102.6 | 158.7 | 63.4 | 63.8 |
| pH | 7.13 | 7.35 | 7.37 | 7.24 | 7.41 | 7.03 | 7.64 | 8.45 | 8.28 |
| Total dissolved solids (TDS) | 256 | 260 | ND | 328 | 297 | 933 | 402 | 521 | ND |
| Mean water depth (cm) | 41 | 60 | 37 | 50 | 40 | 50 | 20 | 20 | 60 |
| Total Nitrogen (N) | 0.28 | 0.00 | 0.00 | 0.00 | 0.00 | 17.41 | 19.32 | 34.56 | 30.59 |
| Total Phosphorous (P) | 0.00 | 0.03 | 0.00 | 0.00 | 0.07 | 0.05 | 0.17 | 0.49 | 0.64 |
| Sediment |  |  |  |  |  |  |  |  |  |
| Organic matter (% w/w) | 1.34 | 1.34 | 0.19 | 0.44 | 0.37 | 0.44 | 0.49 | 0.58 | 0.42 |
| Silt >3.9-63 μm (% w/w) | 4.67 | 4.67 | 0.43 | 1.21 | 3.49 | 6.41 | 2.47 | 2.72 | 3.46 |
| Sand >63-2000 μm (% w/w) | 95.12 | 95.12 | 94.86 | 83.06 | 90.79 | 92.40 | 90.01 | 76.02 | 86.62 |
| Gravel >2000 (% w/w) | 0.23 | 0.23 | 4.70 | 15.72 | 5.71 | 1.20 | 7.53 | 21.27 | 9.91 |
| PPCPs (µg/kg) | | | | | | | | | |
| Amisulpride | bdl | bdl | bdl | bdl | bdl | bdl | bql | 1.52 | 1.13 |
| Amitriptyline | bdl | bdl | bdl | bdl | bdl | bdl | bdl | 2.48 | 1.54 |
| Atenolol | bdl | bdl | bdl | bdl | bdl | bdl | bdl | 1.19 | 1.13 |
| Bisoprolol | bdl | bdl | bdl | bdl | bdl | bdl | bdl | 1.19 | 1.21 |
| Caffeine | bql | bql | bql | bql | bql | 0.20 | 13.1 | 15.9 | 8.64 |
| Citalopram | bdl | bdl | bdl | bdl | bql | bql | bql | 10.95 | 7.67 |
| Propranolol | bdl | bdl | bdl | bdl | bdl | bdl | bdl | 3.14 | 3.09 |
| Sertraline | bdl | bdl | bdl | bdl | bdl | bdl | bdl | 4.58 | 3.41 |
| Tramadol | bdl | bdl | bdl | bdl | bdl | bdl | bql | 20.2 | 22.5 |
| Triclosan | bdl | bdl | bdl | bdl | bdl | bdl | bdl | 1.72 | 1.35 |
| Trimethoprim | bdl | bdl | bdl | 0.07 | 48.0 | bql | bql | bql | bql |
| Venlafaxine | bdl | bdl | bdl | bql | bql | bdl | bql | 12.4 | 10.1 |
| Metals and metalloids (mg/Kg) | | | | | | | | | |
| Li | 1.91 | 1.38 | 0.62 | 1.17 | 1.51 | 1.32 | 2.98 | 3.38 | 3.33 |
| Be | 0.18 | bdl | bdl | bdl | bdl | bdl | 0.29 | 0.52 | bdl |
| Na | bdl | bdl | bdl | bdl | bdl | bdl | 294 | 321 | 220 |
| Mg | 174 | 105 | 35.0 | 292 | 365 | 343 | 4389 | 5012 | 4804 |
| Al | 1341 | 790 | 303 | 662 | 1005 | 984 | 5107 | 6221 | 5508 |
| P | 172 | 253 | 94.7 | 101 | 116 | 118 | 651 | 685 | 827 |
| K | 304 | 212 | bdl | bdl | 207 | 238 | 585 | 745 | 687 |
| Ca | 1122 | 796 | 185 | 36393 | 35934 | 22553 | 40874 | 40602 | 38754 |
| V | 1.54 | 0.85 | 0.33 | 1.85 | 2.29 | 2.35 | 23.0 | 26.6 | 24.1 |
| Cr | 1.71 | 1.07 | bdl | 1.92 | 2.94 | 2.65 | 17.7 | 23.7 | 22.2 |
| Mn | 21.8 | 14.4 | 4.71 | 52.3 | 33.7 | 49.5 | 210 | 192 | 182 |
| Fe | 2762 | 1452 | 660 | 1631 | 2092 | 2225 | 11237 | 12232 | 11466 |
| Co | 0.64 | bdl | bdl | 1.93 | 1.48 | 1.66 | 8.53 | 10.6 | 10.65 |
| Ni | 1.51 | 1.02 | bdl | 1.74 | 2.23 | 2.28 | 21.6 | 26.0 | 25.15 |
| Cu | 3.92 | 4.62 | 0.73 | 3.72 | 4.25 | 5.00 | 11.06 | 19.3 | 20.88 |
| Zn | 11.9 | 9.63 | 2.54 | 9.05 | 15.4 | 15.9 | 50.2 | 84.8 | 102.75 |
| As | 0.88 | bdl | bdl | bdl | bdl | bdl | 0.78 | 0.71 | 0.81 |
| Se | bdl | bdl | bdl | bdl | bdl | bdl | 0.63 | 0.70 | 0.67 |
| Rb | 3.30 | 1.97 | 0.95 | 1.77 | 2.85 | 2.86 | 3.98 | 5.04 | 4.92 |
| Sr | 3.89 | 3.53 | 2.04 | 19.9 | 43.4 | 14.7 | 72.1 | 76.9 | 68.1 |
| Y | 1.20 | 0.62 | 0.32 | 1.20 | 1.31 | 1.38 | 3.43 | 3.77 | 3.74 |
| Mo | bdl | bdl | bdl | bdl | bdl | bdl | 0.22 | 1.34 | 0.25 |
| Ag | 0.05 | bdl | bdl | bdl | bdl | 0.34 | 0.23 | 0.23 | 0.66 |
| Cd | bdl | bdl | bdl | bdl | bdl | bdl | 0.09 | 0.09 | 0.96 |
| In | bdl | bdl | bdl | bdl | bdl | bdl | bdl | bdl | bdl |
| Sn | 0.22 | 0.25 | 0.53 | 0.23 | 0.22 | 0.22 | 0.13 | 0.50 | 0.61 |
| Sb | bdl | bdl | bdl | bdl | bdl | bdl | bdl | bdl | bdl |
| Ba | 13.7 | 10.7 | 30.3 | 11.6 | 834 | 15.0 | 66.1 | 61.4 | 64.0 |
| La | 3.06 | 1.69 | 1.15 | 2.67 | 3.53 | 4.73 | 9.85 | 13.6 | 10.0 |
| Ce | 6.47 | 3.54 | 2.39 | 5.54 | 7.32 | 10.3 | 19.3 | 21.4 | 20.2 |
| Pr | 0.80 | 0.43 | 0.29 | 0.68 | 0.88 | 1.20 | 2.38 | 2.61 | 2.45 |
| Nd | 3.12 | 1.70 | 1.08 | 2.63 | 3.43 | 4.53 | 9.37 | 10.4 | 9.81 |
| Sm | 0.58 | 0.31 | 0.21 | 0.53 | 0.77 | 0.83 | 1.69 | 1.86 | 1.74 |
| Eu | 0.12 | 0.08 | 0.12 | 0.11 | 2.97 | 0.16 | 0.64 | 0.66 | 0.65 |
| Gd | 1.12 | 0.61 | 0.41 | 0.96 | 1.44 | 1.72 | 3.38 | 3.67 | 3.49 |
| Tb | 0.09 | 0.05 | 0.03 | 0.08 | 0.10 | 0.13 | 0.28 | 0.31 | 0.30 |
| Dy | 0.31 | 0.16 | 0.10 | 0.28 | 0.33 | 0.40 | 0.98 | 1.07 | 1.05 |
| Ho | 0.05 | 0.03 | 0.01 | 0.05 | 0.05 | 0.06 | 0.18 | 0.19 | 0.18 |
| Er | 0.13 | 0.07 | 0.03 | 0.11 | 0.13 | 0.14 | 0.41 | 0.44 | 0.43 |
| Tm | 0.02 | 0.01 | 0.01 | 0.02 | 0.02 | 0.02 | 0.07 | 0.07 | 0.06 |
| Yb | 0.09 | 0.04 | 0.01 | 0.08 | 0.08 | 0.10 | 0.28 | 0.30 | 0.29 |
| Lu | bdl | bdl | bdl | bdl | bdl | bdl | 0.05 | 0.04 | 0.04 |
| Pb | 2.08 | 1.32 | 1.09 | 2.67 | 56.38 | 4.98 | 10.28 | 6.32 | 8.08 |
| U | 0.17 | 0.08 | 0.09 | 0.23 | 0.27 | 0.31 | 0.34 | 0.35 | 0.31 |
| PAHs (µg/Kg) | | | | | | | | | |
| Naphthalene (NP) | bdl | bdl | bdl | 2.49 | 2.49 | 3.73 | 1.71 | 2.05 | 2.91 |
| Acenaphthylene (ACY) | bdl | bdl | bdl | bdl | bdl | bdl | bdl | 1.26 | 1.69 |
| Acenaphthene (ACE) | bdl | bdl | bdl | bdl | 1.77 | bdl | 3.86 | bdl | bdl |
| Fluorene (FLU) | bdl | bdl | bdl | bdl | bdl | bdl | 6.66 | 6.13 | bdl |
| Phenanthrene (PHE) | 1.85 | 2.01 | 1.35 | 1.61 | 1.01 | 1.22 | 32.70 | 24.30 | 11.90 |
| Anthracene (ANT) | bdl | bdl | bdl | bdl | bdl | bdl | 5.90 | bdl | bdl |
| Fluoranthene (FLA) | 1.76 | 2.21 | 1.26 | 1.99 | 1.15 | 1.59 | 44.10 | 28.60 | 17.70 |
| Pyrene (PYR) | 3.25 | 3.87 | 2.12 | 2.25 | 1.17 | 1.64 | 48.40 | 52.10 | 25.40 |
| Benzo[a]anthracene (BAA) | bdl | bdl | bdl | 1.34 | bdl | bdl | 16.30 | 1.71 | 4.14 |
| Chrysene (CRY) | bdl | bdl | bdl | 2.15 | 1.52 | 1.73 | 16.30 | 3.42 | 5.50 |
| Benzo[b]fluoranthene (BBF) | bdl | bdl | bdl | 1.60 | 1.00 | 1.33 | 16.30 | 2.87 | 7.04 |
| Benzo[k]fluoranthene (BKF) | bdl | bdl | bdl | bdl | bdl | bdl | 8.19 | 1.64 | 3.50 |
| Benzo[a]pyrene (BAP) | bdl | bdl | bdl | 1.17 | bdl | 1.22 | 23.80 | 2.81 | 8.62 |
| Indeno[1,2,3-c,d]pyrene (IND) | bdl | bdl | bdl | bdl | bdl | bdl | 15.80 | 2.49 | 7.16 |
| Dibenzo[a,h]anthracene (DBAH) | bdl | bdl | bdl | bdl | bdl | bdl | 3.53 | bdl | bdl |
| Benzo[g,h,i]perylene (BGHI) | bdl | bdl | bdl | 1.46 | 1.26 | 1.78 | 18.90 | 3.41 | 9.63 |
| PAHs sum | 6.86 | 8.10 | 4.73 | 16.06 | 11.37 | 14.24 | 262.45 | 132.79 | 105.19 |

bdl – below detection limit; bql – below quantification limit; ND – Not determined (TDS was not determined for WWTPc D2 due to technical problems with the probe in the field). Sediment size classification according to Wentworth, C. K., 1922. A Scale of Grade and Class Terms for Clastic Sediments. The Journal of Geology 30(5): 377-392.

**Table S2.** Pearson correlation values found following correlation analysis within the M, PPCP and PAH whole datasets. Values ≥0.8 determined the pooling of the variables into a joint variable, named metals, PPCPs and PAHs, respectively, that was then integrated in multivariate analyses. *Variables selected as non-colinear for the multivariate analysis.

| **Metals** | | | | | | | | | | | | | | | | | | | | | | | | | | | | | | | | | | | | | | | | | | |
| --- | --- | --- | --- | --- | --- | --- | --- | --- | --- | --- | --- | --- | --- | --- | --- | --- | --- | --- | --- | --- | --- | --- | --- | --- | --- | --- | --- | --- | --- | --- | --- | --- | --- | --- | --- | --- | --- | --- | --- | --- | --- | --- |
|  | **Li** | **Be** | **Na** | **Mg** | **Al** | **P** | **K** | **Ca** | **V** | **Cr** | **Mn** | **Fe** | **Co** | **Ni** | **Cu** | **Zn** | **As** | **Se** | **Rb** | **Sr** | **Y** | **Mo** | **Ag** | **Cd** | **Sn*** | **Ba*** | **La** | **Ce** | **Pr** | **Nd** | **Sm** | **Eu** | **Gd** | **Tb** | **Dy** | **Ho** | **Er** | **Tm** | **Yb** | **Lu** | **Pb** | **U** |
| **Li**  **Be**  **Na**  **Mg**  **Al**  **P**  **K**  **Ca**  **V**  **Cr**  **Mn**  **Fe**  **Co**  **Ni**  **Cu**  **Zn**  **As**  **Se**  **Rb**  **Sr**  **Y**  **Mo**  **Ag**  **Cd**  **Sn**  **Ba**  **La**  **Ce**  **Pr**  **Nd**  **Sm**  **Eu**  **Gd**  **Tb**  **Dh**  **Ho**  **Er**  **Tm**  **Yb**  **Lu**  **Pb**  **U** | 1.000 | 0.677 | 0.918 | 0.948 | 0.972 | 0.947 | 0.980 | 0.622 | 0.950 | 0.956 | 0.924 | 0.970 | 0.943  0.943 | 0.954  0.954 | 0.937 | 0.928  0.928 | 0.839 | 0.942 | 0.963 | 0.885 | 0.967 | 0.692 | 0.681 | 0.599 | 0.286 | -0.089 | 0.939  0.939 | 0.949 | 0.953 | 0.957 | 0.952 | 0.102 | 0.950 | 0.959 | 0.964 | 0.966 | 0.973 | 0.945 | 0.970 | 0.915 | -0.014 | 0.711 |
| **Be** | 0.677 | 1.000 | 0.759 | 0.651 | 0.706 | 0.550 | 0.684 | 0.338 | 0.675 | 0.653 | 0.645 | 0.687 | 0.615 | 0.648 | 0.539 | 0.500 | 0.643 | 0.656 | 0.636 | 0.596 | 0.639 | 0.857 | 0.086 | -0.111 | 0.066 | -0.170 | 0.749 | 0.647 | 0.656 | 0.654 | 0.650 | -0.040 | 0.642 | 0.644 | 0.646 | 0.667 | 0.658 | 0.718 | 0.718 | 0.644 | -0.137 | 0.483 |
| **Na** | 0.919 | 0.759 | 1.000 | 0.980 | 0.978 | 0.930 | 0.906 | 0.644 | 0.984 | 0.966 | 0.972 | 0.979 | 0.956 | 0.974 | 0.874 | 0.872 | 0.713 | 0.983 | 0.823 | 0.912 | 0.946 | 0.767 | 0.573 | 0.450 | 0.320 | -0.163 | 0.956 | 0.941 | 0.946 | 0.948 | 0.940 | 0.030 | 0.939 | 0.949 | 0.955 | 0.967 | 0.961 | 0.983 | 0.955 | 0.980 | -0.091 | 0.690 |
| **Mg** | 0.948 | 0.651 | 0.980 | 1.000 | 0.993 | 0.971 | 0.930 | 0.678 | 0.999 | 0.995 | 0.979 | 0.994 | 0.991 | 0.999 | 0.941 | 0.947 | 0.725 | 0.998 | 0.869 | 0.929 | 0.978 | 0.723 | 0.698 | 0.611 | 0.412 | -0.141 | 0.962 | 0.966 | 0.969 | 0.973 | 0.965 | 0.056 | 0.965 | 0.977 | 0.982 | 0.985 | 0.985 | 0.982 | 0.979 | 0.978 | -0.067 | 0.714 |
| **Al** | 0.972 | 0.706 | 0.978 | 0.993 | 1.000 | 0.967 | 0.960 | 0.648 | 0.994 | 0.993 | 0.967 | 0.997 | 0.983 | 0.994 | 0.947 | 0.945 | 0.773 | 0.991 | 0.908 | 0.918 | 0.980 | 0.757 | 0.681 | 0.580 | 0.385 | -0.140 | 0.974 | 0.970 | 0.974 | 0.977 | 0.969 | 0.056 | 0.968 | 0.979 | 0.984 | 0.987 | 0.989 | 0.981 | 0.981 | 0.965 | -0.068 | 0.715 |
| **P** | 0.948 | 0.550 | 0.930 | 0.971 | 0.967 | 1.000 | 0.935 | 0.562 | 0.966 | 0.967 | 0.931 | 0.968 | 0.955 | 0.974 | 0.948 | 0.953 | 0.748 | 0.975 | 0.856 | 0.858 | 0.938 | 0.633 | 0.739 | 0.711 | 0.442 | -0.197 | 0.898 | 0.918 | 0.922 | 0.928 | 0.912 | -0.006 | 0.915 | 0.935 | 0.943 | 0.948 | 0.951 | 0.927 | 0.942 | 0.952 | -0.128 | 0.591 |
| **K** | 0.980 | 0.684 | 0.906 | 0.930 | 0.960 | 0.935 | 1.000 | 0.542 | 0.933 | 0.942 | 0.893 | 0.952 | 0.917 | 0.938 | 0.931 | 0.919 | 0.806 | 0.925 | 0.964 | 0.852 | 0.940 | 0.715 | 0.708 | 0.572 | 0.317 | -0.092 | 0.940 | 0.944 | 0.946 | 0.949 | 0.940 | 0.095 | 0.943 | 0.948 | 0.946 | 0.942 | 0.951 | 0.918 | 0.945 | 0.893 | -0.019 | 0.690 |
| **Ca** | 0.622 | 0.338 | 0.644 | 0.678 | 0.648 | 0.935 | 0.542 | 1.000 | 0.680 | 0.686 | 0.753 | 0.664 | 0.737 | 0.677 | 0.634 | 0.633 | 0.263 | 0.648 | 0.620 | 0.857 | 0.757 | 0.455 | 0.470 | 0.367 | 0.031 | 0.297 | 0.713 | 0.739 | 0.737 | 0735 | 0.770 | 0.404 | 0.755 | 0.737 | 0.735 | 0.731 | 0.724 | 0.724 | 0.732 | 0.647 | 0.379 | 0.890 |
| **V** | 0.950 | 0.675 | 0.984 | 0.999 | 0.994 | 0.966 | 0.933 | 0.680 | 1.000 | 0.994 | 0.981 | 0.996 | 0.990 | 0.998 | 0.937 | 0.940 | 0.728 | 0.997 | 0.873 | 0.931 | 0.979 | 0.739 | 0.684 | 0.586 | 0.396 | -0.141 | 0.968 | 0.968 | 0.972 | 0.976 | 0.968 | 0.056 | 0.967 | 0.978 | 0.984 | 0.987 | 0.987 | 0.986 | 0.981 | 0.979 | -0.067 | 0.722 |
| **Cr** | 0.956 | 0.653 | 0.966 | 0.995 | 0.993 | 0.967 | 0.942 | 0.686 | 0.994 | 1.000 | 0.965 | 0.990 | 0.994 | 0.997 | 0.966 | 0.967 | 0.719 | 0.990 | 0.895 | 0.930 | 0.981 | 0.757 | 0.714 | 0.627 | 0.445 | -0.117 | 0.973 | 0.969 | 0.972 | 0.976 | 0.969 | 0.079 | 0.968 | 0.968 | 0.984 | 0.983 | 0.985 | 0.971 | 0.979 | 0.954 | -0.046 | 0.728 |
| **Mn** | 0.925 | 0.645 | 0.972 | 0.979 | 0.967 | 0.931 | 0.893 | 0.753 | 0.981 | 0.965 | 1.000 | 0.980 | 0.975 | 0.974 | 0.886 | 0.889 | 0.696 | 0.975 | 0.845 | 0.936 | 0.978 | 0.662 | 0.676 | 0.538 | 0.254 | -0.144 | 0.952 | 0.971 | 0.974 | 0.975 | 0.970 | 0.051 | 0.970 | 0.977 | 0.981 | 0.989 | 0.982 | 0.991 | 0.983 | 0.981 | -0.057 | 0.788 |
| **Fe** | 0.970 | 0.687 | 0.979 | 0.994 | 0.997 | 0.968 | 0.952 | 0.664 | 0.996 | 0.990 | 0.980 | 1.000 | 0.984 | 0.994 | 0.934 | 0.936 | 0.777 | 0.993 | 0.901 | 0.923 | 0.985 | 0.718 | 0.691 | 0.585 | 0.352 | -0.144 | 0.968 | 0.974 | 0.978 | 0.981 | 0.973 | 0.054 | 0.973 | 0.984 | 0.989 | 0.992 | 0.993 | 0.989 | 0.989 | 0.978 | -0.066 | 0.729 |
| **Co** | 0.944 | 0.615 | 0.956 | 0.991 | 0.983 | 0.955 | 0.917 | 0.737 | 0.990 | 0.994 | 0.975 | 0.984 | 1.000 | 0.993 | 0.958 | 0.963 | 0.701 | 0.985 | 0.883 | 0.937 | 0.987 | 0.722 | 0.735 | 0.645 | 0.427 | -0.120 | 0.966 | 0.973 | 0.975 | 0.979 | 0.974 | 0.077 | 0.972 | 0.983 | 0.987 | 0.986 | 0.986 | 0.973 | 0.983 | 0.954 | -0.044 | 0.764 |
| **Ni** | 0.955 | 0.648 | 0.974 | 0.999 | 0.994 | 0.974 | 0.938 | 0.677 | 0.998 | 0.997 | 0.974 | 0.994 | 0.993 | 1.000 | 0.953 | 0.957 | 0.729 | 0.996 | 0.881 | 0.927 | 0.980 | 0.731 | 0.711 | 0.625 | 0.425 | -0.140 | 0.965 | 0.968 | 0.971 | 0.975 | 0.967 | 0.057 | 0.967 | 0.979 | 0.984 | 0.986 | 0.987 | 0.978 | 0.981 | 0.971 | -0.067 | 0.716 |
| **Cu** | 0.938 | 0.539 | 0.874 | 0.941 | 0.947 | 0.948 | 0.931 | 0.634 | 0.937 | 0.966 | 0.886 | 0.934 | 0.958 | 0.953 | 1.000 | 0.993 | 0.681 | 0.931 | 0.907 | 0.859 | 0.940 | 0.731 | 0.786 | 0.733 | 0.531 | -0.127 | 0.926 | 0.925 | 0.925 | 0.931 | 0.921 | 0.058 | 0.921 | 0.937 | 0.939 | 0.930 | 0.938 | 0.887 | 0.932 | 0.862 | -0.065 | 0.676 |
| **Zn** | 0.929 | 0.500 | 0.872 | 0.947 | 0.945 | 0.953 | 0.919 | 0.633 | 0.940 | 0.967 | 0.889 | 0.936 | 0.963 | 0.957 | 0.993 | 1.000 | 0.692 | 0.938 | 0.895 | 0.865 | 0.940 | 0.690 | 0.812 | 0.778 | 0.571 | -0.108 | 0.914 | 0.924 | 0.924 | 0.931 | 0.921 | 0.079 | 0.922 | 0.937 | 0.941 | 0.928 | 0.938 | 0.889 | 0.931 | 0.873 | -0.045 | 0.672 |
| **As** | 0.840 | 0.643 | 0.713 | 0.725 | 0.773 | 0.748 | 0.806 | 0.263 | 0.728 | 0.719 | 0.696 | 0.777 | 0.701 | 0.729 | 0.681 | 0.692 | 1.000 | 0.732 | 0.806 | 0.581 | 0.746 | 0.463 | 0.510 | 0.479 | 0.198 | -0.257 | 0.695 | 0.717 | 0.726 | 0.731 | 0.713 | -0.111 | 0.713 | 0.731 | 0.745 | 0.748 | 0.762 | 0.750 | 0.765 | 0.731 | -0.203 | 0.443 |
| **Se**  **Rb** | 0.942 | 0.656 | 0.983 | 0.998 | 0.991 | 0.975 | 0.925 | 0.648 | 0.997 | 0.990 | 0.975 | 0.993 | 0.985 | 0.996 | 0.931 | 0.938 | 0.732 | 1.000 | 0.853 | 0.916 | 0.968 | 0.717 | 0.685 | 0.605 | 0.419 | -0.165 | 0.952 | 0.955 | 0.959 | 0.963 | 0.953 | 0.031 | 0.953 | 0.967 | 0.974 | 0.979 | 0.978 | 0.978 | 0.971 | 0.983 | -0.093 | 0.683 |
| **Rb** | 0.963 | 0.636 | 0.823 | 0.869 | 0.908 | 0.856 | 0.964 | 0.620 | 0.873 | 0.985 | 0.845 | 0.901 | 0.883 | 0.881 | 0.907 | 0.895 | 0.806 | 0.853 | 1.000 | 0.840 | 0.929 | 0.671 | 0.722 | 0.576 | 0.251 | 0.007 | 0.915 | 0.929 | 0.929 | 0.930 | 0.932 | 0.187 | 0.931 | 0.929 | 0.925 | 0.911 | 0.925 | 0.875 | 0.926 | 0.809 | 0.083 | 0.781 |
| **Sr** | 0.885 | 0.596 | 0.912 | 0.929 | 0.918 | 0.858 | 0.852 | 0.857 | 0.931 | 0.930 | 0.936 | 0.923 | 0.937 | 0.927 | 0.859 | 0.865 | 0.581 | 0.916 | 0.840 | 1.000 | 0.944 | 0.673 | 0.585 | 0.505 | 0.253 | 0.194 | 0.923 | 0.930 | 0.932 | 0.934 | 0.953 | 0.382 | 0.944 | 0.936 | 0.938 | 0.983 | 0.939 | 0.941 | 0.932 | 0.907 | 0.273 | 0.831 |
| **Y** | 0.967 | 0.639 | 0.946 | 0.978 | 0.980 | 0.938 | 0.940 | 0.757 | 0.979 | 0.981 | 0.978 | 0.985 | 0.987 | 0.980 | 0.940 | 0.940 | 0.746 | 0.968 | 0.929 | 0.944 | 1.000 | 0.693 | 0.732 | 0.606 | 0.307 | -0.078 | 0.973 | 0.990 | 0.992 | 0.994 | 0.993 | 0.119 | 0.991 | 0.996 | 0.998 | 0.996 | 0.997 | 0.982 | 0.998 | 0.948 | 0.006 | 0.821 |
| **Mo** | 0.692 | 0.857 | 0.767 | 0.723 | 0.757 | 0.633 | 0.715 | 0.455 | 0.739 | 0.757 | 0.662 | 0.718 | 0.722 | 0.731 | 0.731 | 0.690 | 0.463 | 0.717 | 0.671 | 0.673 | 0.693 | 1.000 | 0.278 | 0.136 | 0.437 | -0.116 | 0.822 | 0.699 | 0.702 | 0.704 | 0.701 | 0.024 | 0.692 | 0.701 | 0.698 | 0.701 | 0.697 | 0.713 | 0.694 | 0.631 | -0.097 | 0.519 |
| **Ag** | 0.681 | 0.086 | 0.573 | 0.698 | 0.681 | 0.739  0.739 | 0.708 | 0.470 | 0.684 | 0.714 | 0.676 | 0.691 | 0.735 | 0.711 | 0.786 | 0.812 | 0.510 | 0.685 | 0.722 | 0.585 | 0.732 | 0.278 | 1.000 | 0.852 | 0.465 | -0.224 | 0.667 | 0.759 | 0.749 | 0.749 | 0.729 | -0.081 | 0.747 | 0.754 | 0.744 | 0.709 | 0.721 | 0.636 | 0.724 | 0.641 | -0.148 | 0.623 |
| **Cd** | 0.599 | -0.111 | 0.450 | 0.611 | 0.580 | 0.711 | 0.572 | 0.367 | 0.586 | 0.627 | 0.538 | 0.585 | 0.645 | 0.625 | 0.733 | 0.778 | 0.479 | 0.605 | 0.576 | 0.505 | 0.606 | 0.136 | 0.852 | 1.000 | 0.627 | -0.099 | 0.480 | 0.569 | 0.566 | 0.577 | 0.561 | 0.018 | 0.568 | 0.592 | 0.601 | 0.574 | 0.596 | 0.501 | 0.585 | 0.545 | -0.057 | 0.334 |
| **Sn*** | 0.286 | 0.066 | 0.320 | 0.412 | 0.385 | 0.442 | 0.317 | 0.031 | 0.396 | 0.445 | 0.254 | 0.352 | 0.427 | 0.425 | 0.531 | 0.571 | 0.198 | 0.419 | 0.251 | 0.253 | 0.307 | 0.437 | 0.465 | 0.627 | 1.000 | -0.184 | 0.337 | 0.292 | 0.290 | 0.301 | 0.276 | -0.116 | 0.280 | 0.313 | 0.321 | 0.289 | 0.309 | 0.263 | 0.287 | 0.292 | -0.215 | -0.008 |
| **Ba*** | -0.089 | -0.170 | -0.163 | -0.141 | -0.140 | -0.197 | -0.092 | 0.297 | -0.141 | -0.117 | -0.144 | -0.144 | -0.120 | -0.140 | -0.127 | -0.108 | -0.257 | -0.165 | 0.007 | 0.194 | -0.078 | -0.116 | -0.224 | -0.099 | -0.184 | 1.000 | -0.096 | -0.089 | -0.092 | -0.091 | -0.023 | 0.980 | -0.043 | -0.094 | -0.099 | -0.121 | -0.099 | -0.114 | -0.120 | -0.163 | 0.993 | 0.170 |
| **La** | 0.939 | 0.749 | 0.956 | 0.962 | 0.974 | 0.898 | 0.940 | 0.713 | 0.968 | 0.973 | 0.952 | 0.968 | 0.966 | 0.965 | 0.926 | 0.914 | 0.695 | 0.952 | 0.915 | 0.923 | 0.973 | 0.822 | 0.667 | 0.480 | 0.337 | -0.096 | 1.000 | 0.981 | 0.982 | 0.982 | 0.980 | 0.097 | 0.979 | 0.980 | 0.977 | 0.974 | 0.973 | 0.968 | 0.974 | 0.918 | -0.021 | 0.815 |
| **Ce** | 0.949 | 0.647 | 0.941 | 0.966 | 0.970 | 0.918 | 0.944 | 0.739 | 0.968 | 0.969 | 0.971 | 0.974 | 0.973 | 0.968 | 0.925 | 0.924 | 0.717 | 0.955 | 0.929 | 0.930 | 0.990 | 0.699 | 0.759 | 0.569 | 0.292 | -0.089 | 0.981 | 1.000 | 0.999 | 0.999 | 0.996 | 0.106 | 0.998 | 0.998 | 0.995 | 0.988 | 0.989 | 0.973 | 0.990 | 0.937 | -0.001 | 0.849 |
| **Pr** | 0.953 | 0.656 | 0.948 | 0.969 | 0.974 | 0.922 | 0.946 | 0.737 | 0.972 | 0.972 | 0.974 | 0.978 | 0.975 | 0.971 | 0.925 | 0.924 | 0.726 | 0.959 | 0.929 | 0.932 | 0.992 | 0.702 | 0.749 | 0.566 | 0.290 | -0.092 | 0.982 | 0.999 | 1.000 | 0.999 | 0.997 | 0.104 | 0.998 | 0.998 | 0.996 | 0.991 | 0.992 | 0.978 | 0.993 | 0.943 | -0.005 | 0.843 |
| **Nd** | 0.957 | 0.654 | 0.940 | 0.973 | 0.977 | 0.928 | 0.949 | 0.735 | 0.976 | 0.976 | 0.975 | 0.981 | 0.979 | 0.975 | 0.931 | 0.931 | 0.731 | 0.963 | 0.930 | 0.934 | 0.994 | 0.704 | 0.749 | 0.577 | 0.301 | -0.091 | 0.982 | 0.999 | 0.999 | 1.000 | 0.997 | 0.105 | 0.998 | 0.999 | 0.997 | 0.993 | 0.994 | 0.979 | 0.994 | 0.946 | -0.005 | 0.835 |
| **Sm** | 0.952 | 0.650 | 0.030 | 0.965 | 0.969 | 0.912 | 0.940 | 0.707 | 0.968 | 0.969 | 0.970 | 0.973 | 0.974 | 0.967 | 0.921 | 0.921 | 0.713 | 0.953 | 0.932 | 0.953 | 0.993 | 0.701 | 0.729 | 0.561 | 0.276 | 0.023 | 0.980 | 0.996 | 0.997 | 0.997 | 1.000 | 0.173 | 0.999 | 0.996 | 0.994 | 0.989 | 0.990 | 0.976 | 0.990 | 0.936 | 0.063 | 0.860 |
| **Eu** | 0.102 | -0.040 | 0.939 | 0.056 | 0.056 | -0.006 | 0.095 | 0.440 | 0.056 | 0.079 | 0.051 | 0.054 | 0.077 | 0.057 | 0.058 | 0.079 | -0.111 | 0.031 | 0.187 | 0.382 | 0.119 | 0.024 | -0.081 | 0.018 | -0.116 | 0.980 | 0.097 | 0.106 | 0.104 | 0.105 | 0.173 | 1.000 | 0.152 | 0.102 | 0.098 | 0.076 | 0.098 | 0.082 | 0.077 | 0.029 | 0.989 | 0.325 |
| **Gd** | 0.950 | 0.642 | 0.949 | 0.965 | 0.968 | 0.915 | 0.943 | 0.755 | 0.967 | 0.968 | 0.970 | 0.973 | 0.972 | 0.967 | 0.921 | 0.922 | 0.713 | 0.953 | 0.931 | 0.944 | 0.991 | 0.692 | 0.747 | 0.568 | 0.280 | -0.043 | 0.979 | 0.998 | 0.998 | 0.998 | 0.999 | 0.152 | 1.000 | 0.997 | 0.994 | 0.988 | 0.989 | 0.974 | 0.990 | 0.937 | 0.044 | 0.856 |
| **Tb** | 0.959 | 0.644 | 0.955 | 0.977 | 0.979 | 0.935 | 0.948 | 0.737 | 0.978 | 0.980 | 0.977 | 0.984 | 0.983 | 0.979 | 0.937 | 0.937 | 0.731 | 0.967 | 0.929 | 0.936 | 0.996 | 0.701 | 0.754 | 0.592 | 0.313 | -0.094 | 0.980 | 0.998 | 0.998 | 0.999 | 0.996 | 0.102 | 0.997 | 1.000 | 0.999 | 0.994 | 0.995 | 0.980 | 0.996 | 0.948 | -0.008 | 0.829 |
| **Dy** | 0.964 | 0.646 | 0.967 | 0.982 | 0.984 | 0.943 | 0.946 | 0.735 | 0.984 | 0.984 | 0.981 | 0.989 | 0.987 | 0.984 | 0.939 | 0.941 | 0.745 | 0.974 | 0.925 | 0.938 | 0.998 | 0.698 | 0.744 | 0.601 | 0.321 | -0.099 | 0.977 | 0.995 | 0.996 | 0.997 | 0.994 | 0.098 | 0.994 | 0.999 | 1.000 | 0.997 | 0.998 | 0.984 | 0.998 | 0.956 | -0.014 | 0.814 |
| **Ho** | 0.966 | 0.667 | 0.961 | 0.985 | 0.987 | 0.948 | 0.942 | 0.731 | 0.987 | 0.983 | 0.989 | 0.992 | 0.986 | 0.986 | 0.930 | 0.928 | 0.748 | 0.979 | 0.911 | 0.938 | 0.996 | 0.701 | 0.709 | 0.574 | 0.289 | -0.121 | 0.974 | 0.988 | 0.991 | 0.993 | 0.989 | 0.076 | 0.988 | 0.994 | 0.997 | 1.000 | 0.998 | 0.991 | 0.998 | 0.967 | -0.036 | 0.796 |
| **Er** | 0.973 | 0.658 | 0.983 | 0.985 | 0.989 | 0.951 | 0.951 | 0.724 | 0.987 | 0.985 | 0.982 | 0.993 | 0.986 | 0.987 | 0.938 | 0.938 | 0.762 | 0.978 | 0.925 | 0.939 | 0.997 | 0.697 | 0.721 | 0.596 | 0.309 | -0.099 | 0.973 | 0.989 | 0.992 | 0.994 | 0.990 | 0.098 | 0.989 | 0.995 | 0.998 | 0.998 | 1.000 | 0.988 | 0.998 | 0.963 | -0.016 | 0.794 |
| **Tm** | 0.945 | 0.718 | 0.955 | 0.982 | 0.981 | 0.927 | 0.918 | 0.724 | 0.986 | 0.971 | 0.991 | 0.989 | 0.973 | 0.978 | 0.887 | 0.889 | 0.750 | 0.978 | 0.875 | 0.941 | 0.982 | 0.713 | 0.636 | 0.501 | 0.263 | -0.114 | 0.968 | 0.973 | 0.978 | 0.979 | 0.976 | 0.082 | 0.974 | 0.980 | 0.984 | 0.991 | 0.988 | 1.000 | 0.987 | 0.979 | -0.031 | 0.782 |
| **Yb** | 0.970 | 0.662 | 0.980 | 0.979 | 0.984 | 0.942 | 0.945 | 0.732 | 0.981 | 0.979 | 0.983 | 0.989 | 0.983 | 0.981 | 0.932 | 0.931 | 0.765 | 0.971 | 0.926 | 0.932 | 0.998 | 0.694 | 0.724 | 0.585 | 0.287 | -0.120 | 0.974 | 0.990 | 0.993 | 0.994 | 0.990 | 0.077 | 0.990 | 0.996 | 0.998 | 0.998 | 0.998 | 0.987 | 1.000 | 0.957 | -0.034 | 0.808 |
| **Lu** | 0.915 | 0.644 | 0.980 | 0.978 | 0.965 | 0.952 | 0.893 | 0.647 | 0.979 | 0.954 | 0.981 | 0.978 | 0.954 | 0.971 | 0.862 | 0.873 | 0.731 | 0.983 | 0.809 | 0.907 | 0.948 | 0.631 | 0.641 | 0.545 | 0.292 | -0.163 | 0.918 | 0.937 | 0.943 | 0.946 | 0.936 | 0.029 | 0.937 | 0.948 | 0.956 | 0.967 | 0.963 | 0.979 | 0.957 | 1.000 | -0.083 | 0.681 |
| **Pb** | 0.915 | -0.137 | -0.091 | -0.067 | -0.068 | -0.128 | -0.019 | 0.379 | -0.067 | -0.046 | -0.057 | -0.066 | -0.044 | -0.067 | -0.065 | -0.045 | -0.203 | -0.093 | 0.083 | 0.273 | 0.006 | -0.097 | -0.148 | -0.057 | -0.215 | 0.993 | -0.021 | -0.001 | -0.005 | -0.005 | 0.063 | 0.989 | 0.044 | -0.008 | -0.014 | -0.036 | -0.016 | -0.031 | -0.034 | -0.083 | 1.000 | 0.267 |
| **U** | 0.711 | 0.483 | 0.690 | 0.714 | 0.715 | 0.591 | 0.690 | 0.890 | 0.722 | 0.728 | 0.788 | 0.729 | 0.764 | 0.716 | 0.676 | 0.672 | 0.443 | 0.683 | 0.781 | 0.831 | 0.821 | 0.519 | 0.623 | 0.334 | -0.008 | 0.170 | 0.815 | 0.849 | 0.843 | 0.835 | 0.860 | 0.325 | 0.856 | 0.829 | 0.814 | 0.796 | 0.794 | 0.782 | 0.808 | 0.681 | 0.267 | 1.000 |

| **PAHs** | | | | | | | | | | | | | | | | | | | | | | | | | | | | |
| --- | --- | --- | --- | --- | --- | --- | --- | --- | --- | --- | --- | --- | --- | --- | --- | --- | --- | --- | --- | --- | --- | --- | --- | --- | --- | --- | --- | --- |
|  | **NP*** | | **ACY*** | | **ACE** | | **FLU** | | **PHE** | | **ANT** | **FLA** | **PYR** | | **BAA** | | **CRY** | | **BBF** | **BKF** | | **BAP** | | **IND** | | **DBAH** | | **BGHI** |
| **NP*** | 1.000 | | 0.333 | | 0.092 | | 0.066 | | 0.136 | | 0.000 | 0.158 | 0.163 | | 0.111 | | 0.244 | | 0.237 | 0.152 | | 0.167 | | 0.156 | | 0.000 | | 0.261 |
| **ACY*** | 0.333 | | 1.000 | | -0.259 | | 0.237 | | 0.400 | | -0.186 | 0.398 | 0.563 | | 0.051 | | 0.130 | | 0.199 | 0.245 | | 0.139 | | 0.242 | | -0.186 | | 0.258 |
| **ACE** | 0.092 | | -0.259 | | 1.000 | | 0.582 | | 0.624 | | 0.900 | 0.655 | 0.453 | | 0.883 | | 0.854 | | 0.815 | 0.774 | | 0.831 | | 0.771 | | 0.900 | | 0.772 |
| **FLU** | 0.066 | | 0.237 | | 0.582 | | 1.000 | | 0.949 | | 0.695 | 0.920 | 0.928 | | 0.713 | | 0.737 | | 0.691 | 0.724 | | 0.688 | | 0.688 | | 0.695 | | 0.663 |
| **PHE** | 0.136 | | 0.400 | | 0.624 | | 0.949 | | 1.000 | | 0.754 | 0.995 | 0.973 | | 0.831 | | 0.858 | | 0.844 | 0.877 | | 0.834 | | 0.853 | | 0.754 | | 0.835 |
| **ANT** | 0.000 | | -0.186 | | 0.900 | | 0.695 | | 0.754 | | 1.000 | 0.786 | 0.583 | | 0.965 | | 0.936 | | 0.910 | 0.901 | | 0.936 | | 0.896 | | 1.000 | | 0.878 |
| **FLA** | 0.158 | | 0.398 | | 0.655 | | 0.920 | | 0.995 | | 0.786 | 1.000 | 0.957 | | 0.869 | | 0.895 | | 0.887 | 0.916 | | 0.877 | | 0.896 | | 0.786 | | 0.881 |
| **PYR** | 0.163 | | 0.563 | | 0.453 | | 0.928 | | 0.973 | | 0.583 | 0.957 | 1.000 | | 0.691 | | 0.733 | | 0.727 | 0.771 | | 0.705 | | 0.743 | | 0.583 | | 0.727 |
| **BAA** | 0.111 | | 0.051 | | 0.883 | | 0.713 | | 0.831 | | 0.965 | 0.869 | 0.691 | | 1.000 | | 0.988 | | 0.983 | 0.977 | | 0.992 | | 0.975 | | 0.965 | | 0.966 |
| **CRY** | 0.244 | | 0.130 | | 0.854 | | 0.737 | | 0.858 | | 0.936 | 0.859 | 0.733 | | 0.988 | | 1.000 | | 0.992 | 0.980 | | 0.990 | | 0.976 | | 0.936 | | 0.981 |
| **BBF** | 0.237 | | 0.199 | | 0.815 | | 0.691 | | 0.844 | | 0.910 | 0.887 | 0.727 | | 0.983 | | 0.992 | | 1.000 | 0.992 | | 0.995 | | 0.992 | | 0.910 | | 0.996 |
| **BKF** | 0.152 | | 0.245 | | 0.774 | | 0.724 | | 0.877 | | 0.901 | 0.916 | 0.771 | | 0.977 | | 0.980 | | 0.992 | 1.000 | | 0.991 | | 0.998 | | 0.901 | | 0.991 |
| **BAP** | 0.167 | | 0.139 | | 0.831 | | 0.688 | | 0.834 | | 0.936 | 0.877 | 0.705 | | 0.992 | | 0.990 | | 0.995 | 0.991 | | 1.000 | | 0.992 | | 0.936 | | 0.989 |
| **IND** | 0.156 | | 0.242 | | 0.771 | | 0.688 | | 0.853 | | 0.896 | 0.896 | 0.743 | | 0.975 | | 0.976 | | 0.992 | 0.998 | | 0.992 | | 1.000 | | 0.896 | | 0.993 |
| **DBAH** | 0.000 | | -0.186 | | 0.900 | | 0.695 | | 0.754 | | 1.000 | 0.786 | 0.583 | | 0.965 | | 0.936 | | 0.910 | 0.901 | | 0.936 | | 0.896 | | 1.000 | | 0.878 |
| **BGHI** | 0.261 | | 0.258 | | 0.772 | | 0.663 | | 0.835 | | 0.878 | 0.881 | 0.727 | | 0.966 | | 0.981 | | 0.996 | 0.991 | | 0.989 | | 0.993 | | 0.878 | | 1.000 |
| **PPCPs** | | | | | | | | | | | | | | | | | | | | | | | | | | | | |
|  | | **Amisulpride** | | **Amitriptyline** | | **Atenolol** | | **Bisoprolol** | | **Caffeine*** | | **Citalopram** | | **Propranolol** | | **Sertraline** | | **Tramadol** | | | **Triclosan** | | **Venlafaxine** | | **Trimethoprim*** | | **Climbazole** | |
| **Amisulpride** | | 1.000 | | 0.995 | | 0.990 | | 0.984 | | 0.738 | | 0.999 | | 0.987 | | 0.999 | | 0.974 | | | 0.999 | | 0.998 | | -0.186 | | 0.999 | |
| **Amitriptyline** | | 0.995 | | 1.000 | | 0.973 | | 0.964 | | 0.749 | | 0.998 | | 0.968 | | 0.995 | | 0.949 | | | 0.992 | | 0.990 | | -0.182 | | 0.991 | |
| **Atenolol** | | 0.990 | | 0.973 | | 1.000 | | 0.999 | | 0.710 | | 0.985 | | 0.999 | | 0.990 | | 0.995 | | | 0.994 | | 0.996 | | -0.189 | | 0.995 | |
| **Bisoprolol** | | 0.984 | | 0.964 | | 0.999 | | 1.000 | | 0.699 | | 0.978 | | 0.999 | | 0.984 | | 0.998 | | | 0.989 | | 0.991 | | -0.189 | | 0.990 | |
| **Caffeine*** | | 0.738 | | 0.749 | | 0.710 | | 0.699 | | 1.000 | | 0.743 | | 0.704 | | 0.738 | | 0.683 | | | 0.733 | | 0.730 | | -0.240 | | 0.731 | |
| **Citalopram** | | 0.999 | | 0.998 | | 0.985 | | 0.978 | | 0.743 | | 1.000 | | 0.982 | | 0.999 | | 0.966 | | | 0.998 | | 0.996 | | -0.185 | | 0.997 | |
| **Propranolol** | | 0.987 | | 0.968 | | 0.999 | | 0.999 | | 0.704 | | 0.982 | | 1.000 | | 0.987 | | 0.997 | | | 0.991 | | 0.994 | | -0.189 | | 0.993 | |
| **Sertraline** | | 0.999 | | 0.995 | | 0.990 | | 0.984 | | 0.738 | | 0.999 | | 0.987 | | 1.000 | | 0.974 | | | 0.999 | | 0.998 | | -0.186 | | 0.999 | |
| **Tramadol** | | 0.974 | | 0.949 | | 0.995 | | 0.998 | | 0.683 | | 0.966 | | 0.997 | | 0.974 | | 1.000 | | | 0.980 | | 0.983 | | -0.188 | | 0.982 | |
| **Triclosan** | | 0.999 | | 0.992 | | 0.994 | | 0.989 | | 0.733 | | 0.998 | | 0.991 | | 0.999 | | 0.980 | | | 1.000 | | 0.999 | | -0.187 | | 0.999 | |
| **Venlafaxine** | | 0.998 | | 0.990 | | 0.996 | | 0.991 | | 0.730 | | 0.996 | | 0.994 | | 0.998 | | 0.983 | | | 0.999 | | 1.000 | | -0.187 | | 0.999 | |
| **Trimethoprim*** | | -0.186 | | -0.182 | | -0.189 | | -0.189 | | -0.240 | | -0.185 | | -0.189 | | -0.186 | | -0.188 | | | -0.187 | | -0.187 | | 1.000 | | -0.187 | |
| **Climbazole** | | 0.999 | | 0.991 | | 0.995 | | 0.990 | | 0.731 | | 0.997 | | 0.993 | | 0.999 | | 0.982 | | | 0.999 | | 0.999 | | -0.187 | | 1.000 | |

**Table S3.** Relative abundance of macroinvertebrates and diatom (%) in samples collected at each sampling site. Up_a,b,c_ correspond to sampling sites immediately upstream the effluent discharge point of WWTP_a,b,c_, D1_a,b,c_ to sampling sites immediately downstream the effluent discharge and D2_a,b,c_ to sites ~500 m downstream the effluent discharge.

|  | **IBMWP Score** | **Upa** | **D1a** | **D2a** | **Upb** | **D1b** | **D2b** | **Upc** | **D1c** | **D2c** |  |
| --- | --- | --- | --- | --- | --- | --- | --- | --- | --- | --- | --- |
| **Macroinvertebrates** |  |  |  |  |  |  |  |  |  |  |  |
| **Ancylidae (ANC)** | 6 | 0.00 | 0.00 | 0.00 | 0.00 | 0.00 | 0.82 | 0.00 | 0.00 | 0.00 |  |
| **Athericidae (ATH)** | 10 | 0.00 | 0.00 | 0.00 | 0.66 | 0.28 | 0.33 | 0.00 | 0.00 | 0.00 |  |
| **Baetidae (BEA)** | 4 | 1.94 | 0.60 | 0.00 | 0.00 | 0.28 | 1.63 | 0.05 | 0.00 | 0.00 |  |
| **Bithyniidae (BIT)** | 3 | 0.00 | 0.00 | 0.00 | 0.00 | 0.00 | 0.33 | 0.00 | 0.00 | 0.00 |  |
| **Caenidae (CAE)** | 4 | 0.00 | 0.00 | 0.00 | 0.00 | 28.13 | 5.55 | 6.40 | 0.00 | 0.00 |  |
| **Ceratopogonidae (CER)** | 4 | 1.46 | 4.79 | 0.00 | 0.66 | 0.28 | 0.16 | 0.00 | 0.47 | 0.83 |  |
| **Chironomidae (CHI)** | 2 | 80.10 | 53.89 | 91.17 | 70.20 | 40.39 | 75.20 | 42.15 | 20.16 | 6.63 |  |
| **Corbiculidae (COR)** | 0 | 11.65 | 12.57 | 0.63 | 0.00 | 0.28 | 0.49 | 0.00 | 0.00 | 0.00 |  |
| **Dendrocoelidae (DEN)** | 5 | 0.00 | 0.00 | 0.00 | 0.00 | 0.00 | 0.00 | 0.10 | 0.00 | 0.00 |  |
| **Dryopidae (DRY)** | 5 | 0.00 | 0.00 | 0.00 | 0.00 | 0.56 | 0.00 | 0.05 | 0.00 | 0.00 |  |
| **Dugesiidae (DUG)** | 5 | 0.00 | 0.00 | 0.00 | 0.00 | 0.00 | 0.00 | 0.19 | 0.00 | 0.00 |  |
| **Ecnomidae (ECN)** | 7 | 0.00 | 0.00 | 0.00 | 0.66 | 0.00 | 0.16 | 0.00 | 0.00 | 0.00 |  |
| **Elmidae (ELM)** | 5 | 0.00 | 0.00 | 0.00 | 9.93 | 0.56 | 0.49 | 0.00 | 0.00 | 0.00 |  |
| **Empididae (EMP)** | 4 | 0.00 | 0.00 | 0.00 | 1.32 | 0.00 | 0.00 | 0.00 | 0.00 | 0.00 |  |
| **Ephemerellidae (EPHE)** | 7 | 0.49 | 0.00 | 0.00 | 0.00 | 0.00 | 0.49 | 0.00 | 0.00 | 0.00 |  |
| **Ephydridae (EPH)** | 2 | 0.00 | 0.00 | 0.00 | 0.66 | 0.28 | 0.00 | 0.00 | 0.00 | 0.00 |  |
| **Erpobdellidae (ERP)** | 3 | 0.00 | 0.00 | 0.00 | 0.00 | 0.00 | 0.00 | 0.05 | 0.00 | 0.00 |  |
| **Gammaridae (GAM)** | 6 | 0.00 | 0.60 | 0.00 | 7.95 | 0.84 | 0.65 | 0.05 | 0.00 | 0.00 |  |
| **Gerridae (GER)** | 3 | 1.46 | 0.00 | 0.00 | 0.00 | 0.00 | 0.00 | 0.00 | 0.00 | 0.00 |  |
| **Glossiphoniidae (GLO)** | 3 | 0.00 | 0.00 | 0.00 | 0.00 | 0.00 | 0.00 | 0.05 | 0.00 | 0.00 |  |
| **Gomphidae (GOM)** | 8 | 0.49 | 1.20 | 0.00 | 0.00 | 0.00 | 0.00 | 0.00 | 0.00 | 0.00 |  |
| **Hydracarina (HYDR)** | 4 | 0.00 | 0.00 | 0.00 | 0.00 | 19.22 | 10.77 | 0.00 | 0.00 | 0.00 |  |
| **Hydrobiidae (HYD)** | 3 | 0.00 | 12.57 | 0.00 | 0.00 | 2.51 | 1.31 | 0.00 | 0.00 | 0.00 |  |
| **Hydropsychidae (HYDRO)** | 5 | 0.00 | 0.00 | 0.00 | 0.66 | 0.28 | 0.82 | 0.00 | 0.00 | 0.00 |  |
| **Libellulidae (LIB)** | 8 | 0.00 | 0.00 | 0.00 | 0.00 | 0.00 | 0.16 | 0.00 | 0.00 | 0.00 |  |
| **Muscidae (MUS)** | 4 | 0.00 | 0.00 | 0.00 | 0.00 | 0.00 | 0.00 | 0.05 | 0.00 | 0.00 |  |
| **Nemathelmintha (NEM)** | 0 | 0.00 | 0.00 | 0.00 | 0.00 | 3.62 | 0.00 | 0.00 | 0.94 | 0.00 |  |
| **Oligochaeta (OLI)** | 1 | 0.00 | 8.98 | 8.20 | 0.00 | 2.51 | 0.00 | 49.50 | 78.44 | 92.27 |  |
| **Ostracoda (OST)** | 3 | 0.00 | 0.00 | 0.00 | 0.00 | 0.00 | 0.00 | 0.24 | 0.00 | 0.00 |  |
| **Physidae (PHY)** | 3 | 0.00 | 0.00 | 0.00 | 0.00 | 0.00 | 0.00 | 1.15 | 0.00 | 0.28 |  |
| **Simuliidae (SIM)** | 5 | 2.43 | 4.79 | 0.00 | 5.30 | 0.00 | 0.16 | 0.00 | 0.00 | 0.00 |  |
| **Tabanidae (TAB)** | 4 | 0.00 | 0.00 | 0.00 | 1.99 | 0.00 | 0.00 | 0.00 | 0.00 | 0.00 |  |
| **Valvatidae (VAL)** | 3 | 0.00 | 0.00 | 0.00 | 0.00 | 0.00 | 0.49 | 0.00 | 0.00 | 0.00 |  |
| **Diatoms** | **IPS score** | | | | | | | | | | |
| ***Achnanthes holsatica* (AHOS)** | 3.8 | 0.00 | 0.00 | 0.40 | 0.00 | 0.00 | 0.00 | 0.00 | 0.00 | 0.00 |  |
| ***Achnanthidium druartii* (ADRU)** | 4.0 | 0.00 | 0.66 | 0.00 | 0.24 | 0.69 | 0.00 | 0.00 | 0.00 | 0.00 |  |
| ***Achnanthidium exiguum var constrictum* (ACEC)** | 4.0 | 0.00 | 0.00 | 0.00 | 0.00 | 0.00 | 0.00 | 0.00 | 6.38 | 15.32 |  |
| ***Achnanthidium minutissimum (ADMI)*** | 5.0 | 71.91 | 66.56 | 60.08 | 3.81 | 32.99 | 31.53 | 0.00 | 0.00 | 0.00 |  |
| ***Achnanthidium minutissimum var affine* (ADMF)** | 5.0 | 0.00 | 0.00 | 0.00 | 1.19 | 1.37 | 0.00 | 0.00 | 0.00 | 0.00 |  |
| ***Amphora inariensis* (AINA)** | 5.0 | 0.00 | 0.00 | 0.00 | 0.00 | 4.12 | 0.00 | 0.00 | 0.00 | 0.00 |  |
| ***Amphora pediculus* (APED)** | 4.0 | 5.93 | 7.58 | 12.50 | 40.29 | 0.00 | 26.13 | 0.00 | 0.00 | 0.00 |  |
| ***Caloneis lancetulla* (CLCT)** | 4.0 | 0.40 | 0.00 | 0.00 | 2.15 | 0.69 | 0.45 | 0.00 | 0.00 | 0.00 |  |
| ***Cocconeis pediculus* (CPED)** | 4.0 | 0.00 | 0.00 | 0.00 | 0.12 | 0.00 | 0.00 | 0.00 | 0.00 | 0.00 |  |
| ***Cocconeis placentula* (CPLA)** | 4.0 | 0.99 | 1.65 | 0.00 | 0.00 | 0.69 | 0.90 | 0.00 | 0.00 | 0.00 |  |
| ***Craticula buderi* (CRAT)** | 2.0 | 0.00 | 0.00 | 0.00 | 0.24 | 0.00 | 0.00 | 0.00 | 0.00 | 0.00 |  |
| ***Cyclotella meneghiniana* (CMEN)** | 2.0 | 0.00 | 0.00 | 0.00 | 0.00 | 0.00 | 0.00 | 20.39 | 4.26 | 0.00 |  |
| ***Cymbella affinis* (CAFF)** | 4.0 | 0.00 | 0.00 | 0.00 | 0.24 | 0.00 | 0.00 | 0.00 | 0.00 | 0.00 |  |
| ***Cymbella lange-bertalotii* (CLBE)** | 5.0 | 0.00 | 0.00 | 0.00 | 0.00 | 0.69 | 0.00 | 0.00 | 0.00 | 0.00 |  |
| ***Cymbella tumida* (CTUM)** | 3.0 | 0.20 | 0.00 | 0.00 | 0.00 | 0.00 | 0.00 | 0.00 | 0.00 | 0.00 |  |
| ***Diploneis oculata* (DOCU)** | 4.0 | 0.00 | 0.00 | 0.00 | 15.97 | 0.69 | 0.00 | 0.00 | 0.00 | 0.00 |  |
| ***Diploneis ovalis* (DOVA)** | 4.0 | 0.00 | 0.00 | 0.00 | 1.31 | 0.00 | 0.00 | 0.00 | 0.00 | 0.00 |  |
| ***Eolimna minima* (EOMI)** | 2.2 | 0.59 | 0.66 | 0.00 | 0.24 | 28.52 | 18.02 | 0.00 | 0.00 | 5.91 |  |
| ***Eolimna subminuscula* (ESBM)** | 2.0 | 0.40 | 0.00 | 0.00 | 0.00 | 0.00 | 0.00 | 0.00 | 0.00 | 28.88 |  |
| ***Fallacia subhamulata* (FSBH)** | 4.0 | 0.20 | 0.00 | 0.00 | 1.67 | 0.00 | 0.00 | 0.00 | 0.00 | 0.00 |  |
| ***Geissleria declivis* (GDCL)** | 4.5 | 0.00 | 0.00 | 0.00 | 0.00 | 0.69 | 0.00 | 0.00 | 0.00 | 0.00 |  |
| ***Gomphonema clavatum* (GCLA)** | 5.0 | 0.00 | 0.00 | 0.00 | 0.00 | 0.00 | 0.23 | 0.00 | 0.00 | 0.00 |  |
| ***Gomphonema italicum* (GITA)** | 4.0 | 0.00 | 0.00 | 0.00 | 0.00 | 0.00 | 0.00 | 0.39 | 10.99 | 0.00 |  |
| ***Gomphonema lagenula* (GLGN)** | 2.0 | 0.00 | 0.00 | 0.00 | 0.00 | 0.00 | 0.00 | 0.00 | 0.00 | 0.00 |  |
| ***Gomphonema micropumilum* (GMPU)** | 5.0 | 0.00 | 0.33 | 0.00 | 0.00 | 4.81 | 0.45 | 0.39 | 0.00 | 0.00 |  |
| ***Gomphonema olivaceum* (GOLI)** | 4.6 | 0.00 | 0.00 | 0.00 | 0.72 | 1.37 | 0.00 | 0.00 | 0.00 | 0.00 |  |
| ***Gomphonema parvulum* (GPAR)** | 2.0 | 0.20 | 0.33 | 0.00 | 0.24 | 0.69 | 0.00 | 14.76 | 0.00 | 3.94 |  |
| ***Gomphonema pumilum* (GPUM)** | 4.5 | 0.59 | 0.33 | 2.42 | 0.00 | 0.00 | 0.00 | 1.55 | 0.00 | 2.63 |  |
| ***Gomphosphenia lingulatiformis* (GPLI)** | 2.0 | 0.00 | 0.00 | 0.00 | 0.00 | 1.37 | 0.00 | 0.00 | 0.00 | 0.00 |  |
| ***Gyrosigma attenuatum* (GYAT)** | 4.0 | 0.00 | 0.00 | 0.00 | 2.03 | 0.00 | 0.00 | 0.00 | 0.00 | 0.00 |  |
| ***Hantzschia amphioxys* (HAMP)** | 1.5 | 0.00 | 0.00 | 0.00 | 0.24 | 0.00 | 0.00 | 0.00 | 0.00 | 0.00 |  |
| ***Karayevia clevei* (KCLE)** | 4.0 | 0.10 | 0.00 | 0.40 | 0.00 | 0.00 | 0.00 | 0.00 | 0.71 | 0.00 |  |
| ***Karayevia kolbei* (KAKO)** | 4.0 | 0.20 | 0.00 | 1.01 | 0.00 | 2.06 | 2.25 | 0.00 | 0.00 | 0.00 |  |
| ***Karayevia oblongella* (KOBG)** | 4.5 | 0.20 | 0.16 | 0.00 | 0.00 | 0.00 | 0.00 | 0.00 | 0.00 | 0.00 |  |
| ***Luticola goeppertiana* (LGOE)** | 2.0 | 0.00 | 0.00 | 0.00 | 0.00 | 3.44 | 1.35 | 0.00 | 0.00 | 0.00 |  |
| ***Luticola saxophila* (LSAX)** | 4.0 | 0.00 | 0.00 | 0.00 | 0.00 | 0.00 | 0.45 | 0.00 | 0.00 | 0.00 |  |
| ***Mayamaea atomus var. permitis* (MAPE)** | 2.3 | 1.38 | 0.00 | 0.00 | 0.00 | 0.00 | 0.00 | 0.00 | 0.00 | 2.63 |  |
| ***Navicula antonii* (NANT)** | 4.0 | 0.00 | 2.31 | 1.61 | 2.38 | 5.50 | 7.21 | 0.00 | 0.00 | 0.44 |  |
| ***Navicula cryptocephala* (NCRY)** | 3.5 | 0.59 | 4.45 | 0.00 | 0.00 | 0.00 | 0.00 | 0.00 | 0.00 | 0.00 |  |
| ***Navicula cryptotenella* (NCTE)** | 4.0 | 3.56 | 0.00 | 2.82 | 0.72 | 0.69 | 1.35 | 0.00 | 2.48 | 0.88 |  |
| ***Navicula gregaria* (NGRE)** | 3.4 | 1.78 | 0.33 | 2.42 | 0.00 | 0.69 | 0.90 | 1.75 | 0.00 | 6.13 |  |
| ***Navicula lanceolata* (NLAM)** | 3.8 | 0.00 | 0.00 | 3.23 | 0.00 | 0.00 | 0.00 | 0.00 | 0.00 | 0.00 |  |
| ***Navicula tripunctata* (NTPT)** | 4.4 | 0.59 | 0.33 | 0.00 | 4.77 | 0.69 | 0.90 | 0.00 | 0.00 | 0.00 |  |
| ***Navicula veneta* (NVEN)** | 2.3 | 0.00 | 0.00 | 7.66 | 0.00 | 0.00 | 0.00 | 1.17 | 0.00 | 2.19 |  |
| ***Nitzschia amphibia* (NAMP)** | 2.0 | 4.35 | 0.49 | 2.42 | 0.00 | 0.34 | 1.80 | 0.78 | 0.00 | 2.19 |  |
| ***Nitzschia dissipata* (NDIS)** | 4.0 | 0.99 | 2.97 | 0.00 | 2.62 | 3.44 | 2.25 | 0.00 | 0.00 | 0.00 |  |
| ***Nitzschia dissipita* (NDME)** | 4.0 | 0.59 | 0.00 | 0.00 | 0.00 | 0.00 | 0.45 | 0.00 | 0.00 | 0.88 |  |
| ***Nitzschia filiformis* (NFIC)** | 3.2 | 1.19 | 3.79 | 0.00 | 0.00 | 0.00 | 0.00 | 0.00 | 74.11 | 0.88 |  |
| ***Nitzschia fonticola* (NFON)** | 3.5 | 0.00 | 0.00 | 0.00 | 0.00 | 0.00 | 0.00 | 53.98 | 0.00 | 20.57 |  |
| ***Nitzschia inconspicua* (NINC)** | 2.8 | 0.00 | 0.82 | 0.20 | 0.00 | 0.69 | 1.13 | 0.39 | 0.00 | 0.00 |  |
| ***Nitzschia solgensis* (NSOL)** | 3.0 | 0.20 | 0.33 | 0.00 | 0.00 | 0.00 | 0.00 | 0.00 | 0.00 | 0.00 |  |
| ***Pinnularia microstauron* (PMIC)** | 2.5 | 0.00 | 0.00 | 0.00 | 0.00 | 0.00 | 0.00 | 0.00 | 0.00 | 0.44 |  |
| ***Planothidium frequentissimum* (PLFR)** | 3.4 | 0.59 | 0.16 | 0.00 | 0.00 | 0.00 | 0.00 | 0.78 | 0.00 | 1.75 |  |
| ***Planothidium lanceolatum* (PTLA)** | 4.6 | 1.48 | 3.46 | 2.82 | 0.00 | 0.34 | 0.90 | 1.94 | 1.06 | 4.38 |  |
| ***Platessa conspicua* (PTCO)** | 4.0 | 0.20 | 0.00 | 0.00 | 0.00 | 0.00 | 0.00 | 0.00 | 0.00 | 0.00 |  |
| ***Platessa hustedtii* (PLHU)** | 4.8 | 0.59 | 0.33 | 0.00 | 0.00 | 0.00 | 0.00 | 0.00 | 0.00 | 0.00 |  |
| ***Rhoicosphenia abbreviate* (RABB)** | 4.0 | 0.00 | 1.98 | 0.00 | 0.24 | 0.00 | 0.00 | 0.00 | 0.00 | 0.00 |  |
| ***Sellaphora pupula* (SPUP)** | 2.6 | 0.00 | 0.00 | 0.00 | 0.00 | 0.00 | 0.00 | 1.75 | 0.00 | 0.00 |  |
| ***Sellaphora raederae* (SRAE)** | 0.0 | 0.00 | 0.00 | 0.00 | 11.68 | 2.06 | 0.90 | 0.00 | 0.00 | 0.00 |  |
| ***Simonsenia delognei* (SIDE)** | 3.0 | 0.00 | 0.00 | 0.00 | 6.91 | 0.00 | 0.00 | 0.00 | 0.00 | 0.00 |  |
| ***Tabularia tabulata* (TTAB)** | 2.0 | 0.00 | 0.00 | 0.00 | 0.00 | 0.00 | 0.45 | 0.00 | 0.00 | 0.00 |  |
| ***Tryblionella apiculata* (TAPI)** | 2.4 | 0.00 | 0.00 | 0.00 | 0.00 | 0.69 | 0.00 | 0.00 | 0.00 | 0.00 |  |

**Table S4.** Variance explained, F value and p-value concerning the testing of the significance of the non-collinear environmental variables in explaining taxa distribution (Monte Carlo permutations), before forward selection procedures towards the production of the reduced RDA (macroinvertebrates) and CCA (phytobenthos) models.

| **Biological dataset** | **Variable** | **Variance explained** | **F value** | **P value** |
| --- | --- | --- | --- | --- |
| **Macroinvertebrates** | Caffeine | 0.872 | 47.876 | **0.0020** |
|  | PAHs | 0.764 | 22.644 | **0.0020** |
|  | TN | 0.756 | 21.670 | **0.0080** |
|  | pH | 0.624 | 11.623 | **0.0140** |
|  | Metals | 0.591 | 10.128 | **0.0120** |
|  | TP | 0.516 | 7.451 | **0.0320** |
|  | PPCPs | 0.450 | 5.735 | 0.0560 |
|  | ACY | 0.389 | 4.466 | 0.0540 |
|  | Sand | 0.351 | 3.789 | 0.0540 |
|  | Gravel | 0.326 | 3.389 | 0.0880 |
|  | O | 0.135 | 1.092 | 0.3380 |
|  | Sn | 0.122 | 0.968 | 0.4300 |
|  | Flow | 0.102 | 0.797 | 0.4000 |
|  | Cond | 0.060 | 0.797 | 0.4000 |
|  | Trime | 0.056 | 0.412 | 0.6220 |
|  | NP | 0.046 | 0.339 | 0.6220 |
|  | Org M | 0.042 | 0.308 | 0.8020 |
|  | Silt | 0.030 | 0.214 | 0.7720 |
|  | Ba | 0.026 | 0.183 | 1.0000 |
| **Diatoms** | Caffeine | 0.878 | 3.348 | **0.0020** |
|  | TN | 0.825 | 3.059 | **0.0040** |
|  | PAHs | 0.778 | 2.815 | **0.0040** |
|  | Metals | 0.764 | 2.742 | **0.0060** |
|  | TP | 0.721 | 2.532 | **0.0120** |
|  | pH | 0.719 | 2.532 | **0.0120** |
|  | ACY | 0.620 | 2.073 | **0.0280** |
|  | PPCPs | 0.600 | 1.987 | **0.0380** |
|  | Sand | 0.571 | 1.865 | **0.0480** |
|  | Gravel | 0.566 | 1.846 | **0.0500** |
|  | NP | 0.488 | 1.535 | 0.1120 |
|  | Flow | 0.434 | 1.333 | 0.2380 |
|  | Sn | 0.346 | 1.023 | 0.4120 |
|  | Org M | 0.345 | 1.021 | 0.3860 |
|  | Cond | 0.341 | 1.005 | 0.4660 |
|  | O | 0.319 | 0.933 | 0.5240 |
|  | Silt | 0.291 | 0.840 | 0.6280 |
|  | Trime | 0.216 | 0.606 | 0.7880 |
|  | Ba | 0.207 | 0.577 | 0.7960 |

**Table S5.** Exploratory correlation exercise to compare the WFD approach to define ecological water quality (EQR values for each site) and the multivariate approach (site scores), based on the macroinvertebrate and the diatom communities. For significant correlations, the P-value was highlighted bold.

| **Community** | **Model** | **Correlated axis** | **Correlation summary** |
| --- | --- | --- | --- |
| Macroinvertebrates | full | Axis 1 | ρ = -0.456; P = 0.218 |
|  |  | Axis 2 | ρ = 0.474; P = 0.198 |
|  | reduced | Axis 1 | ρ = -0.435; P = 0.242 |
|  |  | Axis 2 | ρ = -0.123; P = 0.753 |
| Diatoms | full | Axis 1 | ρ = -0.977; **P= 0.000** |
|  |  | Axis 2 | ρ = -0.229; P = 0.553 |
|  | reduced | Axis 1 | ρ = -0.971; **P = 0,000** |
|  |  | Axis 2 | ρ =; 0.426 P = 0.252 |
